# Effects of combined training performed two or four times per week on 24-h blood pressure, glycosylated hemoglobin and other health-related outcomes in aging individuals with hypertension: Rationale and study protocol of a randomized clinical trial

**DOI:** 10.1371/journal.pone.0251654

**Published:** 2021-05-26

**Authors:** Rodrigo Ferrari, Lucas Betti Domingues, Leandro de Oliveira Carpes, Paula de Azevedo Frank, Vinícius Mallmann Schneider, Sandra C. Fuchs, the GET Study Group

**Affiliations:** 1 Postgraduate Program in Cardiology, Universidade Federal do Rio Grande do Sul, Porto Alegre, RS, Brazil; 2 Physical Education School, Universidade Federal do Rio Grande do Sul, Porto Alegre, RS, Brazil; 3 Sports and Exercise Training Study Group, Hospital de Clínicas de Porto Alegre, Porto Alegre, RS, Brazil; Prince Sattam Bin Abdulaziz University, College of Applied Medical Sciences, SAUDI ARABIA

## Abstract

**Background:**

Acute blood pressure lowering after exercise seems to predict the extent of blood pressure reduction after chronic exercise training interventions. Based on that, the same weekly amount of exercise performed more frequently could be more beneficial for controlling blood pressure.

**Purpose:**

To compare the effects of a combined training program (resistance plus aerobic exercise) performed four or two times per week on 24-h ambulatory blood pressure and other health-related outcomes in middle-aged and older individuals with hypertension.

**Methods:**

This study will be a randomized, parallel group, two-arm, superiority trial. Ninety-eight participants aged 50–80 years with a previous physician diagnosis of hypertension will be randomized to perform two or four sessions per week of combined training using the same total weekly overload. Primary outcomes will be 24-h ambulatory blood pressure and glycosylated hemoglobin; secondary outcomes will be endothelial function, physical fitness and quality of life. The outcomes will be assessed at baseline and at the end of 12 weeks period.

**Results:**

Our conceptual hypothesis is that a combined exercise program performed four or two times per week with equalized weekly volume/overload will improve all outcomes in comparison to the baseline values, and that reductions in 24-h blood pressure and glycosylated hemoglobin will be more pronounced in the group that trained four times a week than twice. The results of this trial are expected to provide evidences to support that higher weekly frequency of combined training should be emphasized in aging adults with hypertension.

## Introduction

Hypertension is one of the most important modifiable risk factors for developing cardiovascular disease, premature mortality [1], and type 2 diabetes [2], with increased prevalence and severity throughout lifespan [3, 4]. Physical independence decreases with advancing age [5, 6] and regular exercise is a cornerstone intervention to improve functionality [7, 8] and reduce blood pressure in aging adults with hypertension [9, 10]. In particular, a combination of resistance and aerobic exercises (i.e., combined training) seems to be the most effective strategy to improve simultaneously cardiovascular and neuromuscular parameters in middle-aged and older individuals [11, 12].

The chronic reduction in blood pressure due to regular exercise seems to result from the sum of the acute decreases that follow each exercise bout (i.e., post-exercise hypotension) [13, 14]. This physiological effect associated with chronic blood pressure reduction may predict the extent of blood pressure lowering after chronic training interventions [15, 16]. Based on this, the same weekly amount of exercise performed more frequently, splitting the total overload into multiple sessions, could be more beneficial for blood pressure control. Although physical exercise guidelines suggest a total weekly volume in minutes (i.e., 150 minutes per week) [17, 18], it’s unclear if the same amount of exercise performed using different weekly frequencies could induce different blood pressure responses. In addition, other important cardiovascular risk factors such as glycemic levels can also be influenced by exercise frequency. Reduction in glycosylated hemoglobin is associated with exercise frequency in type 2 diabetic patients [19], reinforcing the relevance to compare exercise interventions using different weekly training frequencies [20]. Type 2 diabetes and hypertension are closely interlinked because of similar risk factors, such as endothelial dysfunction, vascular inflammation, arterial remodeling, atherosclerosis, dyslipidemia, and obesity [21]. Based on that, performing glycemic assessment in hypertensive individuals is high relevant since older adults with hypertension have greater risk of diabetes developing than normotensive individuals [21].

To the best of our knowledge, no studies have compared the effect of using different weekly combined training frequencies on 24-h ambulatory blood pressure. The aim of the present study is to compare the effects of a combined exercise program performed four versus two times per week on 24-h ambulatory blood pressure, glycosylated hemoglobin and other important health-related outcomes in middle-aged and older individuals with hypertension. The main outcome is the change from baseline to 12 weeks of follow-up in 24-h, daytime, nighttime systolic and diastolic ambulatory blood pressure, and glycosylated hemoglobin between the intervention groups. Secondary outcomes are the difference between mean change in office blood pressure, ambulatory blood pressure variability, endothelial function, as well as cardiorespiratory fitness, muscular strength and quality of life. We anticipate that at 12 weeks, combined exercise program, performed four or two times per week with equalized weekly volume/overload, will improve all outcomes in comparison to the baseline values and these improvements in blood pressure and glycosylated hemoglobin will be more pronounced in the group that trained four times per a week than twice.

## Materials and methods

### Study design

This is a single-center randomized controlled trial with concealed allocation, blinded measurers assessment, with 12 weeks of follow-up analyzed using an intention-to-treat approach. The recruitment will take place between March 2021 and December 2021 through social media and banners. The protocol followed the recommendations for interventional trials (SPIRIT) 2013 guidelines [22].

### Study setting

The study will be conducted in the Clinical Research Center at the Hospital de Clínicas de Porto Alegre (Porto Alegre, RS, Brazil). The training setting is located outside of the center (Porto Alegre, RS, Brazil).

### Participants

Men and women aged 50–80 years will be eligible for this trial if they have:

- Previous physician diagnosis of primary hypertension or taking at least one antihypertensive medication;

- Not engaged in regular exercise programs (3 or more times per week) in the last 3 months before the study.

Among eligible participants, those who fulfill the following criteria will be excluded:

- Physical and muscular injuries that limit to accomplishment of the different training proposed in the study;
- Underlying cardiovascular disease such as acute myocardial infarction, angina, stroke within the last 24 months;
- Presence of heart failure with NYHA classes III or IV;
- Chronic diseases such as cancer, kidney disease requiring dialysis, multiple sclerosis, Parkinson’s disease, among others;
- Body Mass Index ≥ 40 kg/m^2^;
- Diabetes Mellitus with target organs damage;
- Those who did not sign a consent form.

### Interventions

Participants will be randomly enrolled into one of two intervention groups: a combined resistance and aerobic training program performed four times per week (CT4) or two times per week (CT2) throughout 12 weeks. Participants will perform in sequence both resistance and aerobic training on the same session, starting with the resistance exercises immediately followed by the aerobic exercise [23]. Both groups will perform the same total training volume/overload per week (i.e., minutes of exercise per week, number of sets, repetitions, relative intensity, etc.), and only the number of training sessions per week will be different. All participants will be oriented and supervised during the exercise sessions by experienced researchers in order to efficiently perform all exercises. CT4 will perform four combined training sessions per week composed of 10–15 minutes of resistance exercises (1–4 sets of 10–15 repetitions in each exercise, using an intensity corresponding to 50–70% of maximal strength in 3 exercises) followed by 20–25 minutes of aerobic exercise (walking or running at an intensity corresponding to 60–70% of VO_2peak_). CT2 will perform two combined training sessions per week composed of 20–30 minutes of resistance exercise (1–4 sets of 10–15 repetitions, using an intensity corresponding to 50–70% of maximal strength in 6 exercises) followed by 40–50 minutes of aerobic exercise (walking or running at an intensity corresponding to 60–70% of VO_2peak_). As an objective measure of aerobic exercise intensity, heart rate will be continuously recorded with a Polar HR monitor (Polar FT7, Finland). Rating of perceived exertion will be used for determining the intensity of resistance exercises through the Borg scale (CR-10) [24]. The whole combined training periodization is shown in Table 1.

**Table 1:** Combined resistance and aerobic training periodization.

	CT2		CT4			
	Week 1-6	Week 7-12	Week 1-6 (day 1 and 3)	Week 1-6 (day 2 and 4)	Week 7-12 (day 1 and 3)	Week 7-12 (day 2 and 4)
**Resistance exercises**	Sets x Repetitions	Sets x Repetitions	Sets x Repetitions		Sets x Repetitions	Sets x Repetitions
Push-up	2 x 10-12	3 x 10-12	2 x 10-12	-	3 x 10-12	-
Squat	3 x 12-15	4 x 12-15	3 x 12-15	-	4 x 12-15	-
Unilateral balance	1 x 30 s	1 x 45 s	1 x 30 s	-	1 x 45 s	-
Inverted row	2 x 10-12	3 x 10-12	-	2 x 10-12	-	3 x 10-12
Calf raise	2 x 12-15	2 x 18-20	-	2 x 12-15	-	2 x 18-20
Crunch	2 x 15-20	3 x 20	-	2 x 20	-	3 x 20
Volume	20 minutes	30 minutes	10 minutes	10 minutes	15 minutes	15 minutes
Intensity, %1RM	50-60% (4-5 RPE)	60-70% (5-6 RPE)	50-60% (4-5 RPE)	50-60% (4-5 RPE)	60-70% (5-6 RPE)	60-70% (5-6 RPE)
**Aerobic exercise**						
Walking/ Running						
Volume	40 minutes	50 minutes	20 minutes	20 minutes	25 minutes	25 minutes
Intensity, %VO2peak	60-70% (5-6 RPE)	60-70% (5-6 RPE)	60-70% (5-6 RPE)	60-70% (5-6 RPE)	60-70% (5-6 RPE)	60-70% (5-6 RPE)

RPE, rating of perceived exertion; VO2, oxygen consumption; 1RM, one repetition maximum test; CT2, twice a week group; CT4, 4 times per week group.

### Outcome measurements

A blinded member of the research team, unaware of the study-group allocation, will handle the 24-h ambulatory blood pressure data. The primary hypothesis is that CT4 will lower 24-h systolic blood pressure as compared with CT2 and/or reduce glycosylated hemoglobin at a clinically meaningful amount. The outcomes will be measured at baseline and after 12 weeks period.

#### Primary outcomes

The primary outcome will be 24-h, daytime and nighttime systolic blood pressure and glycosylated hemoglobin.

#### Secondary outcomes

Secondary outcomes will include office blood pressure, ambulatory blood pressure variability, endothelial function, quality of life, cardiorespiratory fitness and muscular strength.

Outcomes will be assessed before and after the intervention period, irrespective of attendance or completion status. For participants who dropout of the study at any time after randomization, researchers will use contact information to invite such participants to undergo the end-study outcome assessments (12 weeks after the randomization). International Physical Activity Questionnaire [25] will be applied at the baseline and the end of follow-up in order to assess the level of physical activity for both groups.

### Participant timeline and data collection

**Fig 1:**
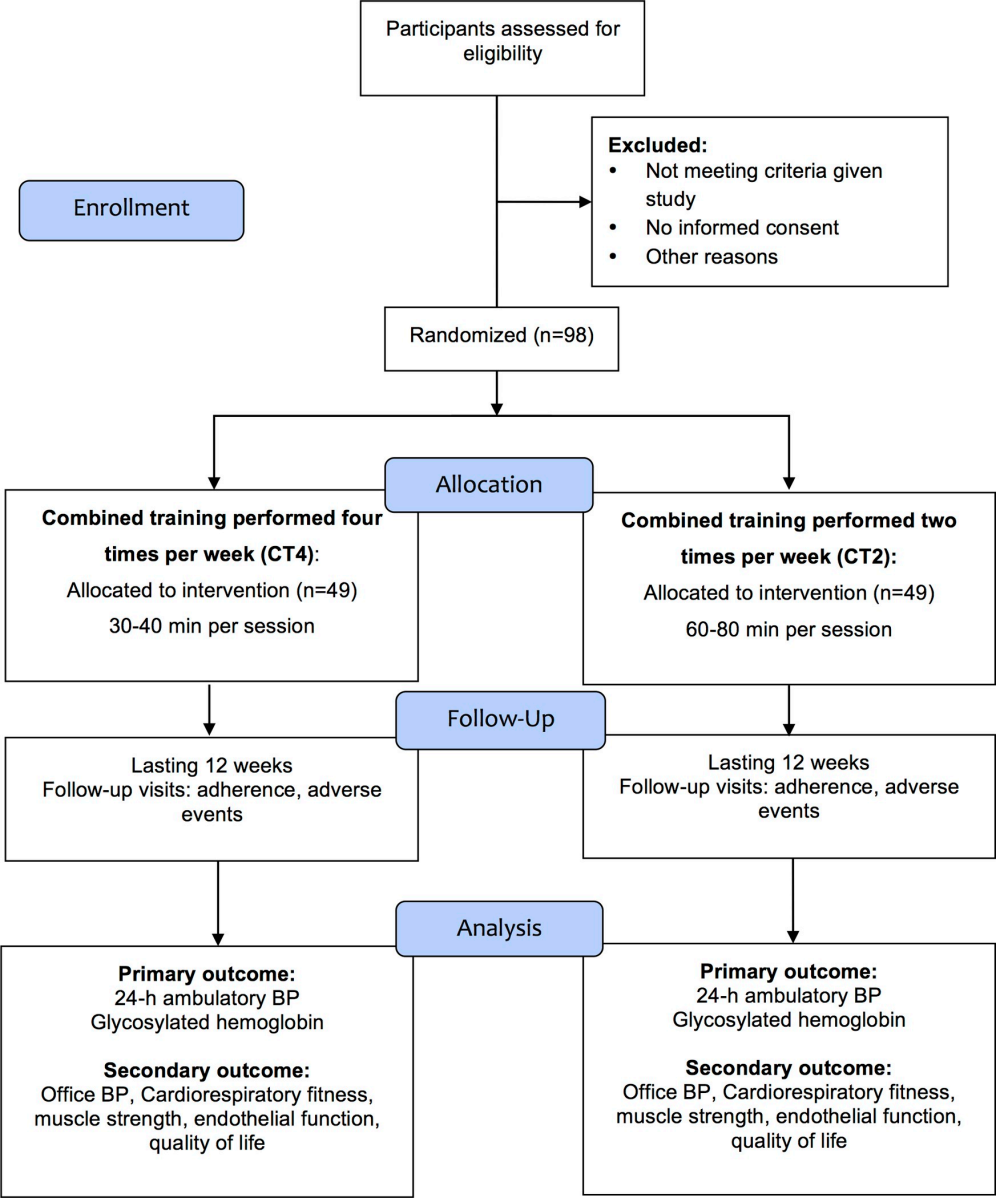
Recruitment and randomized allocation group from the CONSORT 2010 flow diagram. BP, blood pressure.

Participants will be recruited from electronic medical records, phone calls, e-flyers in social media, word of mouth, and personal references. Patients potentially eligible for the study will be contacted by telephone by the trial investigator, who will explain the study and ascertain the patient’s interest to participate. Those who accept to participate will attend the Clinical Research Center of Hospital de Clínicas de Porto Alegre, where the consent form will be signed, and the study assessments will be performed. The schedule of enrollment, interventions, and assessments is presented in Table 2. The allocation of participants and timeline are described below and presented in Fig 1.

**Table 2:** Schedule of enrollment, interventions, and assessments from SPIRIT guidelines.

	STUDY PERIOD			
	Clinical Screning	Allocation	Post-allocation	Close-out
**TIMEPOINT (weeks)**	**-1**	**0**	**1, 4, 8, 12**	**13**
**ENROLLMENT:**				
Eligibility screen	X			
Informed consent	X			
Anthropometric measure	X			X
Allocation		X		
**INTERVENTIONS:**				
Two times per week combined training		X	X	X
Four times per week combined training		X	X	X
**ASSESSMENTS:**				
**Primary outcomes**				
24-h Ambulatory blood pressure	X			X
Glycosilated hemoglobin	X			X
**Secondary outcomes**				
Office blood pressure		X	X	X
Physical fitness		X		X
Endothelial function		X		X
Quality of life		X		X

Those who signed the consent will undergo to anamnesis and clinical screening, with sociodemographic, anthropometric and clinic data assessment, endothelial function evaluation, resting electrocardiogram and questionnaire to evaluate quality of life. After that, participants will perform a familiarization with strength and power muscle tests and with the mask that will be used in the cardiopulmonary test. In the second visit to the clinic, the equipment of ambulatory blood pressure monitoring will be installed. Participants will return to the laboratory to remove the equipment after 24-h and perform evaluations of the cardiorespiratory fitness, muscular strength and power. After that, a venous blood sample will be collected to measure glycosylated hemoglobin in A1C percentage. All variables will be assessed at baseline and at study completion. Participants will be asked to maintain their habitual activities and dietary habits at the time of recruitment and not starting new exercise regimens during the next 12 weeks.

### Measurement of primary outcomes

*Ambulatory blood pressure monitoring* will be assessed throughout 24-h in intervals of 15 minutes in daytime and 20 minutes in nighttime periods. Daytime period starts at 7 AM and nighttime starts at 11 PM, except in case that the participant describes a different sleep time and wake-up time. Participants will receive a diary to note daily activities, symptoms, sleep time and wake-up time, and will be instructed to avoid strenuous physical exercises and alcohol ingestion 24-h prior to exam. Each exam is considered valid when at least 14 measurements during the day and seven measurements at night [26] were successfully performed. If any exam will not be considered valid, a new exam will be conduct within 48-h. Ambulatory blood pressure monitoring will be assessed using an automatic oscillometric device (ABP 2400, Mortara, Milwaukee, EUA).

*Glycosylated hemoglobin* will be assessed through a blood sample with high-performance liquid chromatography technique (Bio-Rad VARIANT II TURBO System, São Paulo, Brazil).

### Measurement of secondary outcomes

*Ambulatory blood pressure variability* will be calculated using the same data recorded in the ambulatory blood pressure device (ABP 2400, Mortara, Milwaukee, EUA). The computer software is programed to calculate the average real variability [27], and the average real variability weighted for the time interval between consecutive readings for both systolic and diastolic blood pressure within the daytime, nighttime, and 24-hour periods [28].

*Office blood pressure* will be measured after 10–20 minutes of rest, with the participant sitting quietly in a chair, according to standardized guidelines [29]. Three measurements, 1–2 minutes apart, will be performed in the arm with the highest initial value. The average of the two last measurements will be used. The participants should avoid caffeine, exercise and smoking for the least 24-h before the measurements. Furthermore, post-exercise hypotension will be measured during intervention period in the first session of weeks 1, 4, 8 and 12. In these sessions, blood pressure will be assessed at the beginning of session (after 10-min rest) and at the end of session in intervals of 15-min, during 60-min. The measures will be taken using a validated automatic oscillometric device (HBP-1100, OMRON Healthcare).

*Endothelial function* will be assessed using the flow-mediated dilatation technique [30]. Baseline longitudinal brachial artery diameters will be measured along with pulsed doppler signals for flow velocity analysis. After baseline recordings are completed, reactive hyperemia will be induced by inflation of a blood pressure cuff to 50 mmHg above previously-measured systolic blood pressure, and cuff inflation will be maintained for 5 min. Two-dimensional images of the brachial artery will be acquired using a linear-array multi-frequency transducer (173 12 MHz) connected to a high-resolution ultrasound system (HD7XE, Phillips, USA). The time of each image acquisition during the cardiac cycle will be determined from simultaneous ECG recording. During image acquisition, anatomical landmarks such as veins and fascial planes should be observed in order to maintain the same image of the artery throughout the study. A high frequency transducer (3–12 MHz) records the dilatation of the brachial artery for 180 s immediately after the release from a 5-min total occlusion maneuver. The longitudinal image of the brachial artery will be recorded continuously for 30 seconds before (baseline image) to up to 3 min after cuff deflation (peak diameter). FMD will be expressed as the percentage change in arterial diameter from baseline: FMD (%) = (peak diameter − baseline diameter) / baseline diameter × 100. All image analyses will be performed offline using the software (Brachial Analyzer, Vascular Tools, Medical Imaging Applications, 181 Coralville, IA USA) by an expert blinded to the sequence of interventions [31].

*Cardiorespiratory fitness* will be assessed using an incremental exercise test on a treadmill in order to determine peak oxygen consumption. The protocol consisted of an initial velocity of 3.5 km/h with 1% inclination for the first 2 minutes. Thereafter, velocity and grade will be incremented by 0.4–0.6 km/h and 0.5–1.0% inclination, respectively, every 1 minute until the participants achieve their volitional exhaustion [32]. The expired gas will be analyzed using a metabolic cart (Metalyzer 3B Cortex, Leipzig, Germany). Blood pressure, electrocardiogram and heart rate will be continuously monitored and recorded throughout the test. The incremental exercise test will be conducted under the direct supervision of a licensed physician.

*Muscular strength* will be assessed through chair-stand test (lower-limbs) and isometric handgrip strength test (upper-limbs). Chair-stand test will be conducted using a folding chair without arms, with a seat height of 43.2 cm. The test will begin with the participant seated in the middle of the chair, back straight, feet approximately shoulder-width apart and placed on the floor at an angle slightly back from the knees. Arms should be crossed at the wrists and held against the chest. At the signal "go", the participant will stand up (body erect and straight) and then return to the initial seated position. The total number of stands executed correctly within 30-s and time to perform the first five repetitions will be used during the analyses [33]. Isometric handgrip strength will be measured in both arms with an analogic hand dynamometer (Jamar Sammons Preston Rolyan, Bolingbrook, IL, USA). The participant will remain seated with upright posture, placing the forearm parallel to the ground (elbow flexed at 90°). Thereafter, the participants will be instructed to perform a maximal squeezing contraction with sustained (isometric) effort lasting 5-s. Three attempts will be performed in each hand with 30-s rest intervals.

*Quality of life* will be evaluated through WHOQOL-BREF questionnaire [34]. The questionnaire contains 26 questions and is divided into four different domains (physical, psychological, social and environmental). The participant will answer the questionnaire alone and researchers provide help only if requested.

### Data management

Data will be collected on standardized forms identified by subject number and trial ID and containing instructions for standardized operational procedures. All data will be stored and managed through the use of Research Electronic Data Capture (REDCap) electronic data capture tools hosted at the center. A researcher will conduct audition for missing or inaccurate data. Data will be backed up daily by automated export procedures from secure servers of the Hospital de Clínicas de Porto Alegre.

### Sample size and power calculation

A sample size of 98 individuals with hypertension on 1:1 ratio (CT2, n = 49) and (CT4, n = 49) allowing a dropout rate of 10%, will be able to detect a difference of 4 ± 10 mmHg in systolic 24-h blood pressure among groups. Statistical power set at 80% including a type I error rate of 5%. WinPepi software calculator was used to estimate the sample size [35].

### Randomization and allocation concealment

The randomization list will be generated by an epidemiologist using web software (www.random.org), with blocks of random sizes that will not be disclosed to ensure concealment. Groups will be generated by stratified randomization per sex, age (50–64 and 65–80 years), and baseline systolic ambulatory blood pressure in order to provide homogeneity on these prognostic factors. The epidemiologist will not participate in the recruitment or assignment to intervention sessions. The outcome assessors and data statistical analyses will be double masking to the endpoints of study. Participants and exercise instructors can’t be blinded for intervention because the nature of it, but they won’t know about randomization until the moment of assignment.

### Strategies of study retention

During the study period, participants allocated to both groups will receive text messages to reinforce time and place of the sessions. We will contact participants to inquire for any adverse events and reschedule missed sessions. Measures of adherence to interventions will be reported as group averages and operationalized as attendance and compliance rates. Attendance is monitored through session’s frequency recording and will be treated as the percent of intervention sessions experienced by a participant given the total number of scheduled sessions (48 sessions for CT4 or 24 sessions for CT2). Adherence will be treated as the percent of intervention sessions fully accomplished without protocol deviations given the total number of scheduled sessions.

### Safety assessments

Data on all adverse events will be collected and included in medical reports, which will be forwarded to the Ethics Committee of the institution. To ensure patient safety, we will monitor any adverse events occurred during the study, and all participants will be monitored throughout the study follow up and during four weeks after study finished.

### Statistical analysis

The endpoints will be analyzed using a full analysis set including all randomized participants, therefore allowing intention-to-treat analyses. A second set of per-protocol analysis will be performed including participants that completed the trial with adherence to at least 80% of the intervention sessions (≥ 38 sessions for participants allocated in the CT4 program, and ≥ 19 sessions for participants allocated to the CT2 program).

Data distribution will be analyzed using Shapiro-wilk test with analysis of histogram and Q-Q plots in combination. The homogeneity of data will be checked using Levene test. Data will be expressed as means and standard error for variables with normal distribution or medians and interquartile range for non-normal distributions and 95% confidence intervals (95%CI). Ambulatory blood pressure monitoring will be analyzed as daytime, nighttime and 24-h systolic and diastolic blood pressure. Primary and secondary outcomes will be analyzed using generalized estimating equation model (GEE) for correlated measures. Adjustment for multiple comparisons will be accomplished using Sequential Bonferroni test. All analysis will be conducted using SPSS Statistics for Windows, version 22.0 (IBM corp., Armonk, NY, USA).

### Ethics approval and consent to participate

All participants will read and sign an informed consent form before enrollment. Participation will be voluntary, and all ethical principles of confidentiality and data protection will be maintained. The study protocol will be conducted according to the principles of the Declaration of Helsinki and in compliance with the Brazilian legal (number 466/2012) and regulatory framework for research involving human beings. The study protocol was approved by the Institutional Review Board of Hospital de Clinicas de Porto Alegre, Brazil (GPPG number 3.641.90), is registered in ClinicalTrials.gov under the identifier: NCT04218903. The expected date for the beginning of the study is March 2021 and anticipated date of completion is December 2021.

### Dissemination policy

We intend to disseminate the methods and findings of the study through individual explanation about the main findings and practical application of the study to each participant; press releases written by journalists and using our personal social media to the general public; and submit scientific manuscripts related to our main findings.

## Discussion

To the best of our knowledge, this study will be the first randomized clinical trial comparing the effects of a combined training program, performed four or twice a week, on blood pressure over 24-h. Other important results related to the health of middle-aged and older individuals and hypertensive patients should be provided.

Different physical activities can be effective to reduce blood pressure in middle-aged and older adults with hypertension [36]. Chronic reductions in blood pressure with regular exercise appear to stem from the summation of acute blood pressure decreases that occur following single bouts of exercise, a phenomenon called post-exercise hypotension [37]. Based on that, the same weekly amount of exercise performed in a higher number of sessions per week could be more beneficial to blood pressure management. Other important aspect is that patients with hypertension are at high risk of type 2 diabetes developing because common aspects of the pathophysiology are shared by both conditions [38]. Combined training is the most effective exercise intervention to improve glycemic control [39] and can also be influenced by exercise frequency [19]. However, there are no data comparing different weekly frequencies of combined training on 24-h blood pressure and glycosylated hemoglobin levels.

Based on the above-mentioned gaps related to the use of the same amount of exercise using different weekly training frequencies and the potential benefits of combined training in aging adults, this study will provide evidence on blood pressure reduction after a combined resistance and aerobic training program performed two and four times per week throughout 12-weeks in middle-aged and older patients with essential hypertension. Besides, other cardiovascular risk factor markers such as glycosylated hemoglobin and physical fitness parameters that can be improved with combined training and could respond differently if performed using more sessions per week will be evaluated in this trial. The results will have implications for the exercise prescription targeted to aging individuals who have essential hypertension.

## Supporting information

S1 Checklist SPIRIT 2013 checklist: Recommended items to address in a clinical trial protocol and related documents*. (DOCX)

S1 File (DOCX)

S2 File (DOCX)
