# Supplementary material for: Effects of combined training performed two or four times per week on 24-h blood pressure, glycosylated hemoglobin and other health-related outcomes in aging individuals with hypertension: Rationale and study protocol of a randomized clinical trial
Source: PLoS One. 2021 May 26;16(5):e0251654. doi: 10.1371/journal.pone.0251654 (PMC8153424; doi:10.1371/journal.pone.0251654)
Supplement: S1 File — (DOCX) [file pone.0251654.s002.docx]

UNIVERSIDADE FEDERAL DO RIO GRANDE DO SUL

**Research Project**

Comparison of different weekly frequencies of combined training on ambulatory blood pressure and other cardiovascular risk factors in individuals with hypertension: a randomized clinical trial

Author:

Rodrigo Ferrari, PhD

Porto Alegre, 2019

**SUMMARY**

[**ABSTRACT 3**](#_Toc62056932)

[**QUALIFICATION OF THE PROBLEM 4**](#_Toc62056933)

[**OBJECTIVES 7**](#_Toc62056934)

[**General Objective 7**](#_Toc62056935)

[**Specific Objectives 7**](#_Toc62056936)

[**MATERIAL AND METHODS 8**](#_Toc62056937)

[**Study Design 8**](#_Toc62056938)

[**Participants recruitment 8**](#_Toc62056939)

[**Experimental procedures 9**](#_Toc62056940)

[**Training programs 9**](#_Toc62056941)

[**Evaluations 10**](#_Toc62056942)

[**Randomization and allocation concealment 14**](#_Toc62056943)

[**Ethical considerations 14**](#_Toc62056944)

[**Statistical analysis 15**](#_Toc62056945)

[**SCHEDULE 16**](#_Toc62056946)

[**FUNDING 17**](#_Toc62056947)

[**REFERENCES 18**](#_Toc62056948)

# ABSTRACT

**Introduction:** Little is known about the weekly frequency required, dissociated from the total weekly training volume, to promote blood pressure (BP) reduction and improvement in other risk markers for cardiovascular diseases. **Objectives:** To evaluate and compare the effects of a combined exercise program (CT) with different weekly frequencies on BP and other cardiovascular risk factors in middle-aged and elderly individuals with hypertension. **Methods:** Men and women aged 50-80 years will participate in this randomized clinical trial with the medical diagnosis of hypertension. They will perform 12 weeks of CT with the same total weekly volume (120-150 min/week). These participants will differentiate their training exclusively by the number of weekly sessions that will be used to perform this weekly volume, one group performing 2 weekly sessions (CT2) and the other group 4 weekly sessions (CT4). Before the beginning of the study (week 0) and at the end of the intervention period (week 13), participants will perform evaluations regarding BP, glycosylated hemoglobin, endothelial function and levels of physical fitness. **Scientific contribution:** This research project is expected to provide solid evidence on the influence of the number of weekly combined training sessions on the chronic reduction of BP and other cardiovascular risk factors in middle-aged and elderly individuals with hypertension.

**Keywords:** Concurrent training; Physical exercise; Ambulatory blood pressure monitoring; Cardiovascular risk factors

# QUALIFICATION OF THE PROBLEM

The sustained elevation of blood pressure (BP) (i.e., systemic arterial hypertension - HTN) assumes a significant condition after the fourth decade of life, with an elevation in the prevalence[^4^](#_ENREF_4) and incidence[^5^](#_ENREF_5) of SAH with increasing age. Sustained elevation of systolic BP (SBP) or diastolic BP (DBP) of 130 or 80 mmHg, respectively[^6^](#_ENREF_6), is associated with increased cardiovascular risk and mortality[^7^](#_ENREF_7)^,^[^8^](#_ENREF_8)^,^ [^9^](#_ENREF_9). Analysis aggregating cohort studies including one million participants has already shown that the risk of coronary artery disease and stroke rise exponentially from PAS 115 mmHg and PAD 75 mmHg[^10^](#_ENREF_10). Furthermore, increases of 10 mmHg and 5 mmHg in SBP and DBP, respectively, are associated with a 40% increase risk of death by stroke and 30% from other cardiovascular diseases (CVD)[^10^](#_ENREF_10).

On the other hand, the reduction of 20 mmHg in SBP and 10 mmHg in DBP leads to a 50% reduction in the chance of cardiovascular events in middle-aged and elderly individuals[^10^](#_ENREF_10). In the Brazilian population, the prevalence of HTN was determined from cross-sectional studies conducted throughout Brazil and according to the criterion greater than or equal to 140/90 mmHg or the use of antihypertensives, with 28.7% (95%IC: 26.2-31.4%)[^11^](#_ENREF_11) in individuals aged 18 to 90 years and 68.9% (95%IC: 64.1-73.3%)[^12^](#_ENREF_12) in individuals aged 60 years or more.

The recent publication of the new directive on prevention, detection, evaluation and management of high blood pressure has established differentiated criteria for the treatment of SAH[^6^](#_ENREF_6). For individuals with no history of CVD and with an estimated risk of the first cardiovascular event (coronary disease, non-fatal myocardial infarction or fatal or non-fatal stroke) of less than 10%, the values of SBP greater than or equal to 140 mmHg or DBP greater than or equal to 90 mmHg were maintained as the cut-off point for starting drug treatment. However, for individuals with previous cardiovascular event, clinical CVD or estimated risk of event greater than or equal to 10%, it established that SBP of 130 mmHg or higher or mean DBP of 80 mmHg or higher should be treated to prevent recurrent events of CVD[^6^](#_ENREF_6). This guideline maintained the previous positions that physical exercise (PE) should be practiced with the objective of reducing BP, and also defined that adult individuals with SBP between 120-139 mmHg or DBP of 80-89 mmHg and without increased cardiovascular risk should follow the non-pharmacological recommendations as a therapeutic strategy. For individuals with prior CVD or high risk, increased physical activity in an PE program is recommended as the first line of treatment[^6^](#_ENREF_6). Similarly, PE programs have shown positive results in other variables associated with increased risk for CVD, with emphasis on results in glycemic control of individuals at higher risk for developing cardiometabolic diseases[^13^](#_ENREF_13)^,^[^14^](#_ENREF_14). If we consider SAH an important risk factor for the development of type II diabetes mellitus[^15^](#_ENREF_15), another clinical condition quite prevalent in the older adult population, the regular practice of PE should be implemented for different populations that are at higher risk of developing CVD.

Due to the high prevalence and low control rates[^16^](#_ENREF_16)^,^[^17^](#_ENREF_17), SAH is considered one of the main modifiable risk factors and one of the most important public health problems[^18^](#_ENREF_18). In addition to the usual treatment of SAH through the use of medications, changes in lifestyle are able to help prevent and treat this condition. Among these changes, the regular practice of PE has received great attention from the scientific community from its beneficial effects in reducing BP[^19-24^](#_ENREF_19). In addition, PE is considered the best strategy for the development of physical fitness, which is directly related to a series of clinically significant outcomes. Different studies have detected an inverse association between cardiorespiratory capacity (i.e., maximum oxygen consumption - VO2max) and risk of death[^25-27^](#_ENREF_25). A cohort study followed 4,631 veteran men with hypertension, demonstrating that greater cardiorespiratory capacity is associated with lower risk of mortality, even in those individuals with other associated risk factors (i.e., diabetes mellitus, body mass index, among others)[^25^](#_ENREF_25). Another variable that is also inversely associated with the risk of death from cardiovascular disease is muscle strength. In men, high values of muscle strength reduced the risk of death by 60% when compared to men with lower muscle strength[^28^](#_ENREF_28).

Results of different meta-analyses confirm the hypotensive effect from performing aerobic exercise (AE)[^22^](#_ENREF_22), resistance (RE)[^29^](#_ENREF_29) and aerobic+resistance (CE)[^24^](#_ENREF_24) in individuals with hypertension. However, the evidence on the benefits of RE is less consistent than that obtained from AE[^3^](#_ENREF_3)^,^ [^24^](#_ENREF_24), which makes necessary to conduct new clinical trials evaluating the chronic benefits of ND in reducing BP in this population. Although considered a physiological response inherent to aging, significant reductions in muscle function, initially observed at ages of 40 years and more pronounced from 65-70 years[^30^](#_ENREF_30), may have serious consequences. One of the main diseases of the muscle system associated with the aging process is Sarcopenia, characterized by loss of strength and muscle mass that leads to a reduction in the functions of this system, leading to an increased risk of adverse outcomes, physical disability, reduced quality of life and increased mortality[^31^](#_ENREF_31). PE, specifically the REs, has been adopted as the main tool to fight this disease. Besides the classic maximum strength gain from this RE model, another variable that has been receiving a growing interest in investigations on the subject is muscle power, pointed out as an important predictor of functional limitations in the elderly[^32^](#_ENREF_32). Due to the different benefits promoted by AE and RE, strategies that associate these two forms of PE are fundamental and should be better investigated in older individuals with higher risk for developing CVD.

Two main mechanisms are associated to the reduction of AP associated to PE: cardiac output (CO) and peripheral vascular resistance (PVR)[^33-35^](#_ENREF_33). Considering that mean BP (MAP) is the functional product of these two variables[^36^](#_ENREF_36), the decrease of one of them, without the proportional increase of the other, results in lower BP values. A recent review pointed out CO as the most responsive mechanism in young individuals and PVR in elderly individuals[^35^](#_ENREF_35). However, from the scarcity of studies evaluating these mechanisms in different populations, new surveys are needed for a better understanding of these responses. In this sense, the analysis of the vascular system, specifically about endothelium-dependent vasodilation is very little explored and may provide new evidence on the mechanisms related to BP reduction through the practice of PE.

The chronic reduction in BP associated with PE seems to be associated with the sum of the acute effects promoted by the exercise sessions (i.e., post-exercise hypotension - PEH). The magnitude and duration of PEH are directly linked to the studied population, presenting better responses in hypertensive individuals, when compared to those who are normotensive[^37^](#_ENREF_37). However, when comparing men and women with similar characteristics, PEH seems to respond in a similar way after performing the same exercise protocol[^38^](#_ENREF_38). When considering this relationship between the acute response (i.e., PEH) and the chronic effect of PE on blood pressure reduction, it is possible to speculate that the number of weekly sessions can have an important influence on the responses of a PE program. This suggests that a greater number of weekly sessions can be more beneficial in reducing BP, if we consider this sum effect of each session that is performed. However, so far, this issue has not been explored in major clinical trials on the subject. Furthermore, the main recommendations on the optimal dose of exercise suggest performing a total weekly volume in minutes per week[^39^](#_ENREF_39)^,^ [^40^](#_ENREF_40), without properly highlighting the importance of weekly frequency decoupled from the total weekly training volume to promote better results in reducing BP and other risk markers for CVDs.

# OBJECTIVES

### General Objective

To evaluate and compare the effects of a combined exercise program (i.e., resistance and aerobic) with different weekly frequencies on BP and other cardiovascular risk factors in individuals with hypertension.

### Specific Objectives

To evaluate and compare the effects of 12 weeks of combined training with different weekly frequencies on the following outcomes in middle-aged and older individuals with hypertension:

*Primary outcomes*

- Daytime, nighttime and 24-hour systolic and diastolic BP, evaluated through ambulatory BP monitoring (ABPM);

- Glycosylated hemoglobin (HbA1c), evaluated through blood collection using the high performance liquid chromatography method.

*Secondary outcomes*

- Endothelial function, evaluated by ultrasonography.

- Cardiorespiratory fitness, evaluated through an ergoespirometric test of maximum effort.

- Neuromuscular function, evaluated through chair-stand test, isometric handgrip test, vertical jump, medicine ball throw and balance tests;

- Quality of life, evaluated through the questionnaire WHOQOL-BREF.

# MATERIAL AND METHODS

# **Study Design**

This is a randomized clinical trial with parallel design in which participants will be allocated to one of two groups: Combined training with two weekly sessions (TC2) and Combined training with four weekly sessions (TC4).

## Participants recruitment

Men and women aged 50-80 will participate in the study with a physician’s diagnosis of hypertension.

Individuals will be eligible for this study if:

a) Accept to participate and sign the informed consent form - TCLE (Suppl 1);

b) Have office blood pressure between 130-179 and 80-110 mmHg for systolic and diastolic blood pressure, respectively, or taking at least one antihypertensive medication;

c) They are clinically able to perform the different training proposed in the study.

Patients who meet the inclusion criteria will be excluded from the study if have:

a) Diseases that limit the performance of physical exercises, such as lung disease, valvular heart disease, renal failure;

b) Underlying cardiovascular disease in the last 24 months such as acute myocardial infarction, angina, stroke or heart failure

c) Diseases that can reduce life expectancy;

d) Regular practitioners of physical activities (i.e., 3 or more sessions per week of moderate or vigorous intensity exercises);

e) BMI ≥39,9 kg/m²;

f) Diabetic proliferative retinopathy.

Participants will be recruited through publicity on social networks and posters placed in the Hospital de Clínicas de Porto Alegre and in nearby establishments. In addition, we will invite individuals registered in databases of previous studies conducted by our research group. Prior to the clinical evaluation of the participants, a brief telephone interview will be conducted in order to identify whether the individuals meet the above criteria and to identify interest in participating in the study. After the telephone contact and the interest manifested, the individuals will perform a clinical evaluation composed of anamnesis, baseline BP measurements, anthropometric evaluation (body mass, height and body mass index), and resting electrocardiogram (ECG) in order to identify eligibility or even cardiovascular diseases that limits the performance of the proposed exercises. In addition, participants will receive a copy of the consent form with the detailed information of the study and those eligible will appear at a pre-established date and time for the other evaluations.

The sample will consist of 98 participants (49 in each intervention), who will be included according to the above criteria. The sample size calculation was performed considering a minimum difference of 4 mmHg, and dispersion (standard deviation) of ± 10 mmHg in systolic and diastolic BP. A statistical power of 80% and an alpha error of P<0.05 were adopted.

## Experimental procedures

The procedures of this study will be conducted at the clinical research center of the Hospital de Clínicas de Porto Alegre (PREVER Study Center and Exercise Pathophysiology Laboratory). From a first telephone contact, individuals interested in participating in the study and who are in agreement with the inclusion and exclusion criteria of the study will be received by the responsible researcher. Once the eligibility of the participant has been confirmed and the participant has answered any questions about the procedures that will be adopted, risks and benefits of the study, the participant will sign the TCLE.

## Training programs

The TC2 and TC4 groups will perform the training with progressive intensity and volume, and will perform the same weekly training overload (i.e., minutes per week, series, repetitions, relative intensity and exercises), but will have this overload divided into different weekly frequencies. The training will last 12 weeks, with a total weekly volume of 120 min in the first weeks (weeks 1-6), progressing to 150 min/week in the last weeks (weeks 7-12). Therefore, the groups will differentiate their training exclusively by the number of weekly sessions that will be used to perform this training, with one group performing 2 weekly sessions and the other group 4 weekly sessions.

The first part of the session will consist of the following RE: push-up, squat, unipodal balance, inverted row, calf raise and crunch. The progression in volume will be performed by increasing the number of sets and repetitions. The intensity of the effort will be adjusted from the range of movement of the exercises, speed of execution, and variations in the number of supports to stabilize the exercises. Individuals must perform 1-4 series of 10-15 repetitions with the load corresponding to 60-70% of 1RM monitored through rating of perceived exertion (RPE), monitored through the CR10 scale. An interval of 90-120 seconds will be adopted between sets and exercises. The second part of the session will consist of AE performed on a treadmill or track and will last 20-50 minutes. The intensity of these sessions will be controlled through the heart rate reserve (HR _reserve_) of the individuals, using intensities between 60-70% of HR _reserve_. In the absence of HR monitors, the stress perception scale corresponding to the above intensities will be used, monitored through the BORG scale with rates of 6-20. In the last session of the week at each 4-week period, after the RE and at the end of each session, information will be recorded regarding the RPE of the participants in the session and the level of enjoyment when conducting the training. It should be noted that the training sessions will always start with the RE, and later on, they will perform the AE, as this seems to be the best order to conduct the training in this population[^41^](#_ENREF_41)^,^ [^42^](#_ENREF_42).

## Evaluations

Before the study begins (week 0) and at the end of the intervention period (week 13), participants will perform evaluations regarding BP, glycated hemoglobin, endothelial function, fitness level, and quality of life. Divided into 3 visits with 24-72h intervals between visits, the different evaluations aim to verify the possible effects of the different interventions on the outcome variables of the study. A food record will be used to characterize eating behavior and to evaluate a possible interference of eating with training responses. The subjects will be recommended not to change their eating habits during the study period.

In the first visit, after signing the TCLE, an anamnesis will be performed to characterize the sample, collection of anthropometric measurements through measures of height and body mass, performed from a stadiometer and an analog balance scale (brand FILIZOLA, BRAZIL). With these values their body mass indexes (BMI) will be calculated, according to the equation mass (kg)/height(m)². A questionnaire will also be carried out to evaluate the quality of life of the participants (WHOQOL-BREF). Also in this session, BP measurements will be performed (after 20 minutes of rest) followed by a familiarization with the exercises that will be used in strength and muscle power tests and with the mask that will be used in gas collection during the cardiopulmonary test.

In the second visit, new BP measures will be performed and the endothelial function evaluation conducted through flow-mediated dilation analysis. The resting electrocardiogram and blood collection will also be performed for analysis of glycated hemoglobin levels. From this blood collection, a sample will be used for analysis of total cholesterol, HDL-c, LDL-c, VLDL-c, which will serve to characterize the sample. Afterwards, the ambulatory blood pressure monitoring equipment (ABPM) will be placed. After 24h of the second visit, each patient must return to the laboratory to remove the ABPM.

On the third visit, after ABPM removal, the evaluation of strength and muscle power tests will be conducted, as well as the cardiorespiratory and unipodal balance evaluation. First the strength, power and unipodal equilibrium tests will be conducted, and after a 10-minute rest, the cardiopulmonary test will be conducted.

*Blood pressure measurement*

Blood pressure during the collection period in the laboratory will be evaluated according to the measurement protocol of the hypertension guidelines of the Brazilian Society of Hypertension and the Brazilian Society of Nephrology. Through these measurements the values of systolic BP, diastolic BP and mean BP will be obtained by the automatic oscillometric method (Dinamap, Critikon, USA). The results of this study will have measures that will be without subjectivity of the operator (operator-independent).

The ABPM, characterized by the periodic measurement of BP in an environment of daily activities, will be performed by portable automatic oscillometric device (ABP 2400, Mortara, Milwaukee, USA). The participants will receive verbal orientations regarding the execution of the exam (operation of the device, measurement position, daily activities). After the end of each experimental session, the cuff of the ABPM device will be placed in the non-dominant arm,, configured to perform the measurement of the daytime every 15 min, and nighttime every 20 min. The waking period will be considered from 6:00 A.M. to 22:59 P.M. and the nighttime period from 11:00 P.M. to 5:59 A.M. All participants will have the beginning of the monitoring scheduled for the same time.

*Glycosylated hemoglobin*

For chronic glycemic data collections, a blood sample will be assessed to evaluate glycosylated hemoglobin (HbA1c) levels with high-performance liquid chromatography method (Bio-rad VARIANT II TURBO System, São Paulo, Brazil).

*Endothelial function*

Flow-mediated dilatation (FMD) of brachial artery will be performed by high resolution ultrasonography (HD7XE, Phillips, USA) in combination with a vascular Doppler (to obtain the flow velocity), through a high frequency transducer (3-12MHz). The evaluation of the FMD will be obtained in a room with low luminosity, controlled temperature and after 15 minutes of lying down. After an arterial occlusion maneuver for 5 minutes, the diameter will be evaluated for 120 seconds and variations will be relativized to the pre-occlusion diameter.

*Cardiorespiratory fitness*

Participants will submitted to maximum pre- and post-training tests to measure maximum oxygen consumption (VO_2máx_). The tests will be performed on ergometric treadmill with mask and gas analyzers, which will be previously connected and calibrated in order to avoid any kind of setback. A 10200 ATL treadmill model from IMBRAMED (Porto Alegre, Brazil) will be used to perform the test, with speed and incline resolution of 0.1km.h^-1^ and 1%, respectively. First it will be heated for 3 minutes and the speed will be slowly increased until it reaches 3 km/h. Soon after, the test will begin at 3-4 km/h and 1% incline, with speed increases of 0.5 km/h every 30 seconds and 1% incline every two minutes. The test will be stopped when the participant indicates their exhaustion by a manual signal. During the tests, participants will be supervised by an experient physician and a researcher. Respiratory gases will be collected through the portable box-type gas analyzer (VO2000, MedGraphics, Ann Arbor, USA).

*Neuromuscular function*

Muscular strength will be assessed through chair-stand test (lower-limbs) and isometric handgrip test (upper-limbs). Isometric handgrip test was chosen because of the great external validity of this evaluation and the association between strength levels obtained in this evaluation and mortality of the population[^28^](#_ENREF_28). Isometric handgrip strength will be measured in both arms with an analogic hand dynamometer (Jamar Sammons Preston Rolyan, Bolingbrook, IL, USA). The participant will remain seated with upright posture, placing the forearm parallel to the ground (elbow flexed at 90°). Thereafter, the participant will be instructed to perform a maximal hand squeezing contraction with sustained (isometric) effort lasting 5-s. Three attempts will be performed in each hand with 30-s rest intervals. The medicine ball throwing test will be performed by 3 attempts with 20 seconds of interval. For this test, a medicine ball of 1 kg for women and 2 kg for men will be used. From a 90 degree bent shoulders position, the participant will throw the ball as far as possible, performing the movement at maximum speed in order to increase the efficiency of the stretching and shortening cycle.

To evaluate the strength and muscle power of the lower limbs will be used the chair-stand test and the countermovement jump test (CMJ), respectively. The evaluation of the muscle power of the lower limbs will be obtained through the height of the CMJ monitored through a software (My Jump 2)^43^. Before starting the data collection the subjects will perform a warm-up that will also serve as a familiarization for the test, performing 3-5 jumps. The subjects will start from the orthostatic position, with their hands on the waist, perform a squat and then the flight phase. For the correct execution of the test, participants will be instructed to keep their hands on their waist throughout the test, perform the transition phase between crouching and the flight phase quickly, jump as high as possible and land at the same starting point. During the test, 3 CMJ jumps will be performed with a 20 second interval between each jump. The strength evaluation of the lower limbs will be obtained by performing the maximum number of stands that the participants can perform for 30 seconds. In addition, the time (seconds) that participants take to perform the first five stands will be counted. The test will start with the participant seated in the chair, the back should not be supported in the stands. Feet should be shoulder-width apart and fully supported on the ground. The upper limbs are crossed at the level of the wrists and against the chest. At the "start" sign, the participant rises to the maximum extension (vertical position) and returns to the initial seated position.

*Quality of life assessment*

The WHOQOL-BREF questionnaire, a version translated and validated in Brazil, will be used to evaluate the quality of life. The questionnaire contains 26 questions and is divided into four domains (physical, psychological, social and enviromental). The answers follow a Likert scale (1 to 5, the higher the score the better the quality of life) and the values of each domain will be expressed in percentage values.

## Randomization and allocation concealment

For the randomization of participants in the different experimental groups (TC2 and TC4), stratified and block randomization will be used. The strata will consist of two age groups (50-65 and 65-80 years). Thus, the participants in the different age groups will be distributed in a balanced way by age stratum and randomly among the different interventions. In addition, participant distribution blocks will be created among the interventions to ensure that the number of participants in each group is equal.

The subjects and researchers responsible for the interventions will be blinded for the allocation of the interventions and will only have access to this information at the time of randomization. The study will have an epidemiologist responsible only for the randomization and confidentiality of the allocation, not participating in the recruitment, evaluation or intervention with the patients. This researcher will inform through telephone contact with the executing researchers the different sequences of randomization for each research subject. The researcher responsible for the statistical analysis of the study outcome variables will also be blinded by different codes for each type of intervention. The randomization process will be performed in online software ([randomization.com](http://www.randomization.com)).

In case of an availability of participants larger than the necessary number will be invited the first participants who get in touch and fulfill all the prerequisites for participation in the study. On the other hand, if availability is lower than that of eligible participants, dissemination of the project will continue until this necessary sample number is reached.

## Ethical considerations

The present study project will be conducted according to the ethical conducts established in Resolution 466/2012, of the National Health Council. The participants will have their copy of the TCLE, which will be clarified by a member of the team of researchers, before the beginning of any procedure in the study. The project will be conducted after approval by the Ethics Committee to which the project will be assigned through the Plataforma Brasil.

During the intervention period, if any osteomuscular pain or discomfort or malaise is reported during the training, it will be stopped immediately. If there is a need for care or procedure that is beyond the competencies of the researcher, they can be performed by the nursing service of the Clinical Research Center (extension 6324) or even by the Emergency Service of the institution. In this case, the researcher will contact him/her by extension 8653 and request contact with the regulating doctor, explain the situation and accompany the patient to the Emergency. The individual will be moved by wheelchair, stretcher or ambulance. In addition, the researchers responsible will contact a relative or close person (reported by the participant in the first visit), to inform about what happened.

## Statistical analysis

Shapiro-Wilk and Levene tests will be used for data normality and variance homogeneity, respectively. If the data present normal and homogeneous distribution, parametric statistics will be used. Transformations will be performed if the data do not present normal distribution to make them parametric. The Generalized Estimating Equations (GEE) and the Bonferroni post-hoc test will be used for the comparison between the moments and groups/ interventions. In addition, statistical analysis by protocol will be performed, in which those who have presented three consecutive absences in training will be excluded from the analysis, as well as those who obtain a frequency lower than 75% during the period of interventions. Statistical analysis by intention to treat will also be performed, in which all randomized participants will be included in the analyses. The level of significance adopted will be α = 0.05 for all analyses. The statistical package SPSS version 22.0 will be used to perform the statistical procedures.

# SCHEDULE

|  | 2019/2 | 2020/1 | 2020/2 | 2021/1 |
| --- | --- | --- | --- | --- |
| Submission of the project to research ethics committee | x |  |  |  |
| Training of researchers | x |  |  |  |
| Recruitment of participants | x | x | x |  |
| Evaluations and interventions | x | x | x | X |
| Analysis and discussion of results |  |  |  | X |
| Writing and submission of scientific article |  |  |  | X |

# FUNDING

Permanent materials such as blood pressure monitors, equipments for exercise sessions, as well as other permanent materials needed for project evaluations are now available at the Exercise Physiopathology Laboratory of Hospital de Clínicas de Porto Alegre and at the center of the PREVER study.

|  | Quantity | Unitary value | Total value |
| --- | --- | --- | --- |
| A4 paper (package) | 5 | R$ 13,00 | R$ 65,00 |
| Ultrasound gel (300g) | 10 | R$3,24 | R$ 32,40 |
| ECG electrode | 1000 | R$ 0,63 | R$ 360,00 |
| Rest electrocardiogram | 98 | R$ 5,15 | R$ 504,70 |
| Glycosylated hemoglobina (blood sample) | 196 | R$ 10,17 | R$ 1993,32 |
| Total cholesterol (blood sample) | 196 | R$ 1,85 | R$ 362,60 |
| Ergospirometry | 98 | R$ 30,00 | R$ 2940,00 |
| **TOTAL** |  |  | **R$ 6.258,02** |

# REFERENCES

**1.** Ferrari R, Kruel LF, Cadore EL, et al. Efficiency of twice weekly concurrent training in trained elderly men. *Experimental gerontology*. 2013; 48: 1236-42.

**2.** Ferrari R, Fuchs SC, Kruel LF, et al. Effects of Different Concurrent Resistance and Aerobic Training Frequencies on Muscle Power and Muscle Quality in Trained Elderly Men: A Randomized Clinical Trial. *Aging and disease*. 2016; 7: 697-704.

**3.** Ferrari R, Umpierre D, Vogel G, et al. Effects of concurrent and aerobic exercises on postexercise hypotension in elderly hypertensive men. *Experimental gerontology*. 2017; 98: 1-7.

**4.** da Costa JS, Barcellos FC, Sclowitz ML, et al. Hypertension prevalence and its associated risk factors in adults: a population-based study in Pelotas. *Arq Bras Cardiol*. 2007; 88: 59-65.

**5.** Moreira LB, Fuchs SC, Wiehe M, Gus M, Moraes RS and Fuchs FD. Incidence of hypertension in Porto Alegre, Brazil: a population-based study. *J Hum Hypertens*. 2008; 22: 48-50.

**6.** Whelton PK, Carey RM, Aronow WS, et al. 2017 ACC/AHA/AAPA/ABC/ACPM/AGS/APhA/ASH/ASPC/NMA/PCNA Guideline for the Prevention, Detection, Evaluation, and Management of High Blood Pressure in Adults: A Report of the American College of Cardiology/American Heart Association Task Force on Clinical Practice Guidelines. *J Am Coll Cardiol*. 2017.

**7.** Bundy JD, Li C, Stuchlik P, et al. Systolic Blood Pressure Reduction and Risk of Cardiovascular Disease and Mortality: A Systematic Review and Network Meta-analysis. *JAMA Cardiol*. 2017; 2: 775-81.

**8.** Burnier M, Oparil S, Narkiewicz K and Kjeldsen SE. New 2017 American Heart Association and American College of Cardiology guideline for hypertension in the adults: major paradigm shifts, but will they help to fight against the hypertension disease burden? *Blood Press*. 2018; 27: 62-5.

**9.** Oparil S, Acelajado MC, Bakris GL, et al. Hypertension. *Nat Rev Dis Primers*. 2018; 4: 18014.

**10.** Lewington S, Clarke R, Qizilbash N, Peto R, Collins R and Prospective Studies C. Age-specific relevance of usual blood pressure to vascular mortality: a meta-analysis of individual data for one million adults in 61 prospective studies. *Lancet*. 2002; 360: 1903-13.

**11.** Picon RV, Fuchs FD, Moreira LB, Riegel G and Fuchs SC. Trends in prevalence of hypertension in Brazil: a systematic review with meta-analysis. *PloS one*. 2012; 7: e48255.

**12.** Picon RV, Fuchs FD, Moreira LB and Fuchs SC. Prevalence of hypertension among elderly persons in urban Brazil: a systematic review with meta-analysis. *American journal of hypertension*. 2013; 26: 541-8.

**13.** Ishiguro H, Kodama S, Horikawa C, et al. In Search of the Ideal Resistance Training Program to Improve Glycemic Control and its Indication for Patients with Type 2 Diabetes Mellitus: A Systematic Review and Meta-Analysis. *Sports medicine*. 2016; 46: 67-77.

**14.** Delevatti RS, Kanitz AC, Alberton CL, et al. Glucose control can be similarly improved after aquatic or dry-land aerobic training in patients with type 2 diabetes: A randomized clinical trial. *J Sci Med Sport*. 2016; 19: 688-93.

**15.** Kirkman MS, Briscoe VJ, Clark N, et al. Diabetes in older adults. *Diabetes Care*. 2012; 35: 2650-64.

**16.** Fuchs FD, Gus M, Moreira WD, et al. Blood pressure effects of antihypertensive drugs and changes in lifestyle in a Brazilian hypertensive cohort. *Journal of hypertension*. 1997; 15: 783-92.

**17.** Gus I, Harzheim E, Zaslavsky C, Medina C and Gus M. Prevalence, awareness, and control of systemic arterial hypertension in the state of Rio Grande do Sul. *Arquivos brasileiros de cardiologia*. 2004; 83: 429-33; 4-8.

**18.** Sociedade Brasileira de C, Sociedade Brasileira de H and Sociedade Brasileira de N. [VI Brazilian Guidelines on Hypertension]. *Arquivos brasileiros de cardiologia*. 2010; 95: 1-51.

**19.** Sillanpaa E, Hakkinen A, Nyman K, et al. Body composition and fitness during strength and/or endurance training in older men. *Medicine and science in sports and exercise*. 2008; 40: 950-8.

**20.** Sillanpaa E, Hakkinen A, Punnonen K, Hakkinen K and Laaksonen DE. Effects of strength and endurance training on metabolic risk factors in healthy 40-65-year-old men. *Scandinavian journal of medicine & science in sports*. 2009; 19: 885-95.

**21.** Moraes MR, Bacurau RF, Casarini DE, et al. Chronic conventional resistance exercise reduces blood pressure in stage 1 hypertensive men. *Journal of strength and conditioning research / National Strength & Conditioning Association*. 2012; 26: 1122-9.

**22.** Cornelissen VA, Buys R and Smart NA. Endurance exercise beneficially affects ambulatory blood pressure: a systematic review and meta-analysis. *Journal of hypertension*. 2013; 31: 639-48.

**23.** Millar PJ, McGowan CL, Cornelissen VA, Araujo CG and Swaine IL. Evidence for the role of isometric exercise training in reducing blood pressure: potential mechanisms and future directions. *Sports medicine*. 2014; 44: 345-56.

**24.** Cornelissen VA and Smart NA. Exercise training for blood pressure: a systematic review and meta-analysis. *Journal of the American Heart Association*. 2013; 2: e004473.

**25.** Kokkinos P, Doumas M, Myers J, et al. A graded association of exercise capacity and all-cause mortality in males with high-normal blood pressure. *Blood pressure*. 2009; 18: 261-7.

**26.** Blair SN, Kampert JB, Kohl HW, 3rd, et al. Influences of cardiorespiratory fitness and other precursors on cardiovascular disease and all-cause mortality in men and women. *Jama*. 1996; 276: 205-10.

**27.** Lee DC, Artero EG, Sui X and Blair SN. Mortality trends in the general population: the importance of cardiorespiratory fitness. *Journal of psychopharmacology*. 2010; 24: 27-35.

**28.** Ruiz JR, Sui X, Lobelo F, et al. Association between muscular strength and mortality in men: prospective cohort study. *Bmj*. 2008; 337: a439.

**29.** MacDonald HV, Johnson BT, Huedo-Medina TB, et al. Dynamic Resistance Training as Stand-Alone Antihypertensive Lifestyle Therapy: A Meta-Analysis. *Journal of the American Heart Association*. 2016; 5.

**30.** American College of Sports M, Chodzko-Zajko WJ, Proctor DN, et al. American College of Sports Medicine position stand. Exercise and physical activity for older adults. *Medicine and science in sports and exercise*. 2009; 41: 1510-30.

**31.** Sayer AA, Robinson SM, Patel HP, Shavlakadze T, Cooper C and Grounds MD. New horizons in the pathogenesis, diagnosis and management of sarcopenia. *Age and ageing*. 2013; 42: 145-50.

**32.** Reid KF and Fielding RA. Skeletal muscle power: a critical determinant of physical functioning in older adults. *Exercise and sport sciences reviews*. 2012; 40: 4-12.

**33.** Hagberg JM, Montain SJ and Martin WH, 3rd. Blood pressure and hemodynamic responses after exercise in older hypertensives. *Journal of applied physiology*. 1987; 63: 270-6.

**34.** Rezk CC, Marrache RC, Tinucci T, Mion D, Jr. and Forjaz CL. Post-resistance exercise hypotension, hemodynamics, and heart rate variability: influence of exercise intensity. *European journal of applied physiology*. 2006; 98: 105-12.

**35.** Brito LC, Queiroz AC and Forjaz CL. Influence of population and exercise protocol characteristics on hemodynamic determinants of post-aerobic exercise hypotension. *Brazilian journal of medical and biological research.*2014; 47: 626-36.

**36.** MacDonald JR. Potential causes, mechanisms, and implications of post exercise hypotension. *Journal of human hypertension*. 2002; 16: 225-36.

**37.** Queiroz AC, Sousa JC, Cavalli AA, et al. Post-resistance exercise hemodynamic and autonomic responses: Comparison between normotensive and hypertensive men. *Scandinavian journal of medicine & science in sports*. 2015; 25: 486-94.

**38.** Queiroz AC, Rezk CC, Teixeira L, Tinucci T, Mion D and Forjaz CL. Gender influence on post-resistance exercise hypotension and hemodynamics. *International journal of sports medicine*. 2013; 34: 939-44.

**39.** Borjesson M, Onerup A, Lundqvist S and Dahlof B. Physical activity and exercise lower blood pressure in individuals with hypertension: narrative review of 27 RCTs. *Br J Sports Med*. 2016; 50: 356-61.

**40.** Piercy KL, Troiano RP, Ballard RM, et al. The Physical Activity Guidelines for Americans. *Jama*. 2018; 320: 2020-8.

**41.** Cadore EL, Izquierdo M, Alberton CL, et al. Strength prior to endurance intra-session exercise sequence optimizes neuromuscular and cardiovascular gains in elderly men. *Experimental gerontology*. 2012; 47: 164-9.

**42.** Pinto SS, Alberton CL, Bagatini NC, et al. Neuromuscular adaptations to water-based concurrent training in postmenopausal women: effects of intrasession exercise sequence. *Age*. 2015; 37: 9751.

**43.** Balsalobre-Fernández, Carlos et al. “The validity and reliability of an iPhone app for measuring vertical jump performance.” Journal of sports sciences 33 15 (2015): 1574-9.

**SUPLL 1 - Consent form**

**Project title:** Comparison of different weekly frequencies of combined training on ambulatory blood pressure and other cardiovascular risk factors in individuals with hypertension: a randomized clinical trial

Regular physical training is an important way to benefit the health of healthy or sick people. Health entities recommend the practice of physical activity for prevention and as part of the treatment of hypertension, and it can also improve the quality of life of practitioners.

You are being invited to participate in a research whose objective is to evaluate the effects of combined training (muscle strengthening exercises and walking or running exercises) on 24-hour blood pressure reduction (ABPM). All participants will perform 12 weeks of combined training with the same weekly volume of training (i.e. the same amount of minutes per week). However, they will be divided randomly as to the number of weekly sessions they will have to complete this volume. Half of the participants will exercise twice a week and the other half four times a week.

This research is being conducted by the Exercise Physiopathology Laboratory of the Clinical Research Center of the Hospital de Clínicas de Porto Alegre (HCPA) in partnership with the PREVER study center, both located at the Hospital de Clínicas de Porto Alegre, RS.

If you agree to participate in the research, the procedures involved in your participation are as follows:

Evaluations

Visit 1

- Your height, body mass and waist circumference will be measured.

- Your blood pressure will be measured through an automatic monitor.

- A blood collection will be performed to evaluate the levels of glycated hemoglobin and total cholesterol.

- A resting electrocardiogram will be performed.

This visit will be carried out at the Clinical Research Center of HCPA and will last 2 hours.

Visit 2

- The brachial artery dilation will be evaluated by imaging examination (ultrasonography). In addition, we will place the ambulatory blood pressure monitoring (ABPM) equipment for 24 hours. You should return to the laboratory after 24 hours to remove the equipment.

This visit will last 30 minutes.

Visit 3

- Removal of ABPM.

- Cardiorespiratory evaluations, strength and muscle power and balance in one foot will be performed. You may feel fatigue in the minutes following the tests, moderate pain and/or muscle fatigue in the legs during the 24 -72h after the tests.

After the preliminary evaluations, you will be drawn to join one of the study training groups:

Group 1: Combined training twice a week - In this group participants perform muscle strengthening training, using body weight as an overload; aerobic training, walking or running on a treadmill lasting 40 minutes per session evolving up to 50 minutes per session at the end of the study.

Group 2: Combined training four times a week - Participants in this group perform muscle and aerobic strength training, as described above, lasting 20 minutes per session evolving up to 25 minutes per session at the end of the study.

The combined training program consists of strength and walking/running exercises, with different weekly frequencies (twice or four times a week), for 12 weeks. The trainings will be performed in the physical exercise laboratory of the Hospital de Clínicas de Porto Alegre, where you will be accompanied by physical education professionals and/or physicians. The sessions will begin with strength exercises: push up, squat, inverted row, unilateral balance, calfrise and crunch; after that, walking and/or running exercises will be done. After the execution of the follow-up programs, the same evaluations made during the initial visits will be repeated. That is, you must return to the Clinical Research Center of HCPA for 3 more visits, in which the procedures described above (Visits 1-3) will be repeated.

You may or may not benefit from participating in the research. It is hoped that both programs can positively alter your blood pressure profile, but this may not be true for everyone participating in the study, as there is great variation between the responses of each. However, the study of participants' responses to the programs can make a contribution to understanding hypertension in the population.

We will have safety measures and procedures for risk prevention during the research procedures. In case of emergency, the medical service will be immediately contacted. Researchers will provide first aid assistance, and the person you informed in case of emergency will be notified. In case of any occurrence or damage resulting from your participation in the research, you will receive all necessary assistance at no personal cost. In case any new information appears that makes your participation in the study unfeasible, harmful or modified, you will be notified in advance. In case of abnormalities appearing in any of the exams performed, you will be advised to seek medical treatment.

Your participation in the research is totally voluntary, it is not mandatory. In case you decide not to participate, or even to give up participating and withdraw your consent, there will be no damage to the service you receive or may receive in the institution. Changes of any nature in the study procedures will be informed to you before they occur and you will be given all the autonomy to decide your stay in the study.

No payment is expected for your participation in the research and you will have no cost with respect to the procedures involved. The information obtained from your participation will be treated anonymously. The data will be available to the participant and to whom you authorize, and may be used anonymously for academic scientific purposes.

If you have any questions, ask as many questions as you wish before deciding your participation. If you have any questions, you may contact the responsible researcher, Prof. Dr. Rodrigo Ferrari da Silva at (51) 999012660, or the Research Ethics Committee of the Hospital de Clínicas de Porto Alegre (HCPA), at (51) 33597640, or at the 2nd floor of HCPA, room 2227, from Monday to Friday, from 8 A.M. to 5 P.M.

This consent form is signed in two copies, one for the participant and the other for the researchers.

__________________________________________ (Name of research participant)

__________________________________________ (Signature)

__________________________________________ (Name of researcher)

__________________________________________ (Signature)

Porto Alegre, _____ de __________________________ de ___________.
