# Supplementary material for: Effects of combined training performed two or four times per week on 24-h blood pressure, glycosylated hemoglobin and other health-related outcomes in aging individuals with hypertension: Rationale and study protocol of a randomized clinical trial
Source: PLoS One. 2021 May 26;16(5):e0251654. doi: 10.1371/journal.pone.0251654 (PMC8153424; doi:10.1371/journal.pone.0251654)
Supplement: S2 File — (DOCX) [file pone.0251654.s003.docx]

UNIVERSIDADE FEDERAL DO RIO GRANDE DO SUL

**Projeto de Pesquisa**

Comparação de diferentes frequências semanais de treinamento combinado na pressão arterial e outros fatores de risco cardiovasculares em indivíduos com hipertensão: um ensaio clínico randomizado

Autor:

Rodrigo Ferrari, PhD

Porto Alegre, 2019

**SUMÁRIO**

[**RESUMO 3**](#_Toc16682741)

[**QUALIFICAÇÃO DO PROBLEMA 4**](#_Toc16682742)

[**OBJETIVOS 8**](#_Toc16682743)

[**Objetivo Geral 8**](#_Toc16682744)

[**Objetivos Específicos 8**](#_Toc16682745)

[**MATERIAL E MÉTODOS 9**](#_Toc16682746)

[**Delineamento do Estudo 9**](#_Toc16682747)

[**Amostra 9**](#_Toc16682748)

[**Procedimentos experimentais 10**](#_Toc16682749)

[**Treinamentos aplicados 10**](#_Toc16682750)

[**Avaliações 11**](#_Toc16682751)

[**Randomização e sigilo de alocação 15**](#_Toc16682752)

[**Considerações éticas 16**](#_Toc16682753)

[**Análise estatística 16**](#_Toc16682754)

[**CRONOGRAMA 18**](#_Toc16682755)

[**ORÇAMENTO 19**](#_Toc16682756)

[**REFERÊNCIAS 20**](#_Toc16682757)

# RESUMO

**Introdução**: Pouco se sabe sobre a frequência semanal necessária, dissociada do volume de treinamento semanal total, para promover redução de pressão arterial (PA) e melhora em outros marcadores de risco para doenças cardiovasculares. **Objetivos**: Avaliar e comparar os efeitos de um programa de exercícios físicos combinados (i.e., resistido e aeróbio - TC) com diferentes frequências semanais sobre a PA e outros fatores de risco cardiovasculares em indivíduos de meia-idade e idosos com hipertensão. **Métodos:** Participarão deste ensaio clínico randomizado, em paralelo, homens e mulheres com idade entre 50-80 anos, com o diagnóstico médico de hipertensão. Eles realizarão 12 semanas de TC com o mesmo volume total semanal (120-150 min/semana). Esses participantes diferenciarão seus treinamentos exclusivamente pelo número de sessões semanais que serão utilizadas para realizar esse volume semanal, um grupo realizando 2 sessões semanais (TC2) e o outro grupo 4 sessões semanais (TC4). Antes do início do estudo (semana 0) e ao final do período de intervenções (semana 13), os participantes realizarão avaliações referentes a PA, hemoglobina glicada, função endotelial e níveis de aptidão física. **Contribuição científica:** Espera-se que o presente projeto de pesquisa forneça evidência sólida sobre a influência do número de sessões semanais de treinamento combinado na redução crônica de PA e outros fatores de risco cardiovasculares em indivíduos de meia-idade e idosos com hipertensão.

**Palavras-chave:** Treinamento concorrente; Exercício físico; Monitoramento ambulatorial de pressão arterial; Fatores de risco cardiovascular

# QUALIFICAÇÃO DO PROBLEMA

A elevação sustentada da pressão arterial (PA) (i.e., hipertensão arterial sistêmica – HAS) assume condição marcante a partir da quarta década de vida, havendo elevação na prevalência^[4](#_ENREF_4)^ e incidência^[5](#_ENREF_5)^ de HAS com o aumento da idade. A elevação sustentada da PA sistólica (PAS) ou diastólica (PAD) a partir de 130 ou 80 mmHg, respectivamente^[6](#_ENREF_6)^, associa-se a elevação do risco cardiovascular e da mortalidade^[7](#_ENREF_7),[8](#_ENREF_8), [9](#_ENREF_9)^. Análise agregando estudos de coorte incluindo um milhão de participantes já havia mostrado que o risco de doença arterial coronariana e acidente vascular cerebral elevam-se exponencialmente a partir de PAS 115 mmHg e PAD 75 mmHg^[10](#_ENREF_10)^. Ainda, aumentos de 10 mmHg e 5 mmHg na PAS e na PAD, respectivamente, estão associadas com aumento de 40% no risco de morte por acidente vascular cerebral e 30% por morte por outras doenças cardiovasculares (DCVs)^[10](#_ENREF_10)^. Por outro lado, a redução de 20 mmHg na PAS e 10 mmHg na PAD, acarreta redução de 50% na chance de eventos cardiovasculares em indivíduos de meia-idade e idosos^[10](#_ENREF_10)^. Na população brasileira, a prevalência de HAS foi determinada a partir de estudos transversais realizados em todo o Brasil e segundo o critério de PA maior ou igual a 140/90 mmHg ou uso de anti-hipertensivos, sendo 28.7% (95%IC: 26.2-31.4%)^[11](#_ENREF_11)^ em indivíduos com 18 a 90 anos e 68.9% (95%IC: 64.1-73.3%)^[12](#_ENREF_12)^ em indivíduos com 60 anos ou mais.

A recente publicação da nova diretriz de prevenção, detecção, avaliação e manejo da PA elevada estabeleceram critérios diferenciados para o tratamento da HAS^[6](#_ENREF_6)^. Para indivíduos sem história de DCV e com uma estimativa de risco de primeiro evento cardiovascular (doença coronariana, infarto do miocárdio não fatal ou acidente vascular cerebral fatal ou não fatal) inferior a 10%, manteve os valores de PAS maior ou igual a 140 mmHg ou PAD maior ou igual a 90 mmHg como ponto de corte para iniciar o tratamento medicamentoso. Contudo, para indivíduos com evento cardiovascular prévio, DCV clínica ou risco estimado de evento maior ou igual a 10%, estabeleceu que PAS de 130 mmHg ou superior ou PAD média de 80 mmHg ou superior devem ser tratados para prevenir eventos recorrentes de DCV^[6](#_ENREF_6)^. Essa diretriz não só manteve as posições prévias estabelecendo que exercício físico (EF) deva ser praticado com o objetivo de reduzir a PA, como definiu que indivíduos adultos com PAS entre 120-139 mmHg ou PAD de 80-89 mmHg e sem risco cardiovascular aumentado deveriam ater-se as recomendações não farmacológicas como estratégia terapêutica. Para indivíduos com DCV prévia ou risco elevado, o aumento da atividade física em um programa de EF é recomendado como primeira linha de tratamento^[6](#_ENREF_6)^. Da mesma forma, programas de EF tem apresentado resultados positivos em outras variáveis associadas ao aumento de risco para DCV, com destaque para os resultados no controle glicêmico de indivíduos que apresentam maior risco para o desenvolvimento de doenças cardiometabólicas^[13](#_ENREF_13),[14](#_ENREF_14)^. Se considerarmos a HAS um importante fator de risco para o desenvolvimento de diabetes *mellitus* tipo II^[15](#_ENREF_15)^, outra condição clínica bastante prevalente na população adulta mais velha, a prática regular de EF deve ser implementada para diferentes populações que estão sob maior risco de desenvolver DCV.

Em virtude da alta prevalência e das baixas taxas de controle^[16](#_ENREF_16),[17](#_ENREF_17)^, a HAS é considerada um dos principais fatores de risco modificáveis e um dos mais importantes problemas de saúde pública^[18](#_ENREF_18)^. Além do tratamento usual da HAS feito através do uso de medicamentos, mudanças no estilo de vida são capazes de auxiliar na prevenção e tratamento dessa condição. Dentre essas mudanças, a prática regular de exercícios físicos (EF) vem recebendo grande atenção por parte da comunidade científica a partir dos seus efeitos benéficos na redução da PA^[19-24](#_ENREF_19)^. Além disso, o EF é considerado a melhor estratégia para o desenvolvimento da aptidão física, que está diretamente relacionada a uma série de desfechos clínicamente significativos. Diferentes pesquisas detectaram associação inversa entre a capacidade cardiorrespiratória (i.e., consumo máximo de oxigênio - VO_2max_) e o risco de morte^[25-27](#_ENREF_25)^. Um estudo de coorte, acompanhou 4.631 homens veteranos com hipertensão, demonstrou que maior capacidade cardiorrespiratória está associada a menor risco de mortalidade, mesmo naqueles indivíduos com outros fatores de risco associados (i.e., diabetes *mellitus*, índice de massa corporal, entre outros)^[25](#_ENREF_25)^. Outra variável que também associa-se inversamente com o risco de morte por doença cardiovascular é a força muscular. Em homens, valores elevados de força muscular reduziram em 60% o risco de morte, quando comparado com homens com menor força muscular ^[28](#_ENREF_28)^.

Resultados de diferentes metanálises confirmam o efeito hipotensor a partir da realização de exercício aeróbio (EA)^[22](#_ENREF_22)^, resistido (ER)^[29](#_ENREF_29)^ e aeróbio+resistido (EC)^[24](#_ENREF_24)^ em indivíduos com hipertensão. Entretanto, as evidências sobre os benefícios do EC são menos consistentes que as obtidas a partir do EA^[3](#_ENREF_3), [24](#_ENREF_24)^, o que torna necessário a realização de novos ensaios clínicos avaliando os benefícios crônicos do EC na redução da PA dessa população. Embora considerada uma resposta fisiológica inerente ao envelhecimento, as reduções significativas na função muscular, inicialmente observadas a partir de 40 anos e mais pronunciadas a partir dos 65-70 anos^[30](#_ENREF_30)^, podem trazer sérias consequências. Uma das principais doenças do sistema muscular associada ao processo de envelhecimento é a Sarcopenia, caracterizada pela perda de força e massa muscular que leva a redução das funções desse sistema, levando a um aumento de risco de desfechos adversos, incapacidade física, redução da qualidade de vida e aumento de mortalidade^[31](#_ENREF_31)^. O EF, especificamente os ERs, vem sendo adotados como principal ferramenta para combater essa doença. Além do clássico ganho de força máxima a partir desse modelo de EF, outra variável que vêm recebendo um crescente interesse nas investigações sobre o tema é a potência muscular, apontada como importante preditor de limitações funcionais no idoso^[32](#_ENREF_32)^. Em função dos diferentes benefícios promovidos pelo EA e ER, estratégias que associem essas duas formas de EF são fundamentais e devem ser melhor investigadas em indivíduos mais velhos e com maior risco para o desenvolvimento de DCVs.

Dois principais mecanismos estão associados à redução de PA associada ao EF: o débito cardíaco (DC) e a resistência vascular periférica (RVP)^[33-35](#_ENREF_33)^. Considerando que a PA média (PAM) é o produto funcional dessas duas variáveis^[36](#_ENREF_36)^, a diminuição de uma delas, sem o aumento proporcional da outra, resulta em menores valores de PA. Uma revisão recente apontou o DC como o mecanismo mais responsivo em indivíduos jovens e a RVP em indivíduos idosos^[35](#_ENREF_35)^. Entretanto, a partir da escassez de estudos avaliando esses mecanismos em diferentes populações, novas pesquisas são necessárias para um melhor entendimento dessas respostas. Nesse sentido, a análise do sistema vascular, especificamente sobre a vasodilatação endotélio-dependente é muito pouca explorada e pode fornecer novas evidências sobre os mecanismos relacionados a redução de PA através da prática de EF.

A redução crônica da PA associada ao EF parece estar associada ao somatório dos efeitos agudos promovidos pelas sessões de exercício (i.e., hipotensão pós-exercício - HPE). A magnitude e a duração HPE estão diretamente ligadas à população estudada, apresentando melhores respostas em indivíduos hipertensos, quando comparado aqueles normotensos^[37](#_ENREF_37)^. Entretanto, ao compararmos homens e mulheres com características semelhantes, a PA parece responder de maneira similar após a realização de um mesmo protocolo de exercícios ^[38](#_ENREF_38)^. Ao considerarmos essa relação entre a resposta aguda (i.e.,HPE) e o efeito crônico do EF na redução de pressão arterial, é possível especular que o número de sessões semanais pode exercer importante influência nas respostas de um programa de EF. Isso sugere que um maior número de sessões semanais pode ser mais benéfico na redução de PA, se considerarmos esse efeito somatório de cada sessão que é realizada. Entretanto, até o presente momento, essa questão não foi explorada nos principais ensaios clínicos sobre o tema. Ainda, as principais recomendações sobre a dose ideal de EF sugerem a realização de um volume semanal total de EF em minutos por semana^[39](#_ENREF_39), [40](#_ENREF_40)^, sem destacar devidamente a importância da frequência semanal dissociada do volume de treinamento semanal total para promover melhores resultados na redução de PA e outros marcadores de risco para DCVs.

# OBJETIVOS

### Objetivo Geral

Avaliar e comparar os efeitos de um programa de exercícios físicos combinados (i.e., resistido e aeróbio) com diferentes frequências semanais sobre a PA e outros marcadores de risco cardiovascular em indivíduos com hipertensão.

### Objetivos Específicos

Avaliar e comparar os efeitos de 12 semanas de treinamento combinado com diferentes frequências semanais sobre os seguintes desfechos em indivíduos de meia-idade e idosos com hipertensãos.

*Desfechos primários*

- Monitoramento de PAS e PAD nos períodos diurno, noturno e ao longo de 24 horas, avaliadas através da monitorização ambulatorial de PA (MAPA);

- Hemoglobina Glicada (HbA1c), avaliada através de coleta sanguínea utilizando o método da Cromatografia Líquida de Alta Performance.

*Desfechos secundários*

- Função endotelial, avaliada através de ultrassonografia.

- Aptidão cardiorrespiratória, avaliada através de um teste ergoespirométrico de esforço máximo.

- Função neuromuscular, avaliada através dos testes de preensão manual, sentar e levantar, salto vertical, arremesso de *medicine ball* e teste de equilíbrio unipodal;

- Qualidade de vida, avaliada através de questionário WHOQOL-BREF.

# MATERIAL E MÉTODOS

# **Delineamento do Estudo**

Trata-se de um ensaio clínico randomizado, em paralelo, no qual os participantes serão alocados para um de dois grupos: Treinamento combinado com duas sessões semanais (TC2) e Treinamento combinado com quatro sessões semanais (TC4).

## Amostra

Participarão do estudo homens e mulheres com idade entre 50-80 anos, com o diagnóstico médico de hipertensão. Serão elegíveis para o presente estudo os indivíduos que:

a) Aceitem participar e assinem termo de consentimento livre e esclarecido - TCLE (ANEXO 1);

b) Apresentem valores de PA de consultório entre 130-179 e 80-110 mmHg ou em uso de pelo menos um medicamento anti-hipertensivo;

c) Estejam clinicamente aptos à realização dos diferentes treinamentos propostos no estudo.

Serão excluídos do estudo aqueles pacientes que, preenchendo os critérios de inclusão, apresentem:

a) Doenças que limitem a realização de exercícios físicos, como doença pulmonar, doença cardíaca valvular, insuficiência renal;

b) Doença cardiovascular subjacente diagnosticada previamente por médico, ocorrida nos últimos 24 meses, como infarto agudo do miocárdio, angina ou acidente vascular cerebral, ou insuficiência cardíaca;

c) Doenças que reduzam a expectativa de vida;

d) Praticantes regulares de atividades físicas (i.e., 3 ou mais sessões por semana de exercícios de intensidade moderada ou vigorosa);

e) Índice de massa corporal maior que 39,9 kg/m²;

f) Retinopatia proliferativa diabética.

Os participantes serão recrutados através de divulgação nas redes sociais e cartazes colocados no HCPA e em estabelecimentos próximos. Além disso, nós convidaremos indivíduos cadastrados em bancos de dados de estudos prévios realizados pelo nosso grupo de pesquisa. Antes da avaliação clínica dos participantes, será realizada uma breve entrevista por telefone a fim de identificar se os indivíduos se encaixam nos critérios supracitados e identificar o interesse em participar do estudo. Após o contato telefônico e o interesse manifestado, os indivíduos realizarão uma avaliação clínica composta de anamenese, medidas basais de PA, avaliação antropométrica (massa corporal, estatura e índice de massa corporal), e eletrocardiograma em repouso (ECG), a fim de identificar confirmar a elegibilidade ou ainda alguma limitação cardiovascular que impessa a realização dos exercícios propostos. Além disso, receberão cópia do consentimento com as informações detalhadas do estudo e os elegíveis comparecerão em data e horários pré-estabelecidos para as demais avaliações.

A amostra será constituída por 98 participantes (49 em cada intervenção), os quais serão incluídos de acordo com os critérios supracitados. O cálculo amostral foi realizado considerando uma diferença mínima de 4 mmHg, e dispersão (desvio padrão) de ± 10 mmHg na PAS ou PAD. Foi adotado um poder estatístico de 80% e um erro alfa de P<0,05.

## Procedimentos experimentais

Os procedimentos do presente estudo serão conduzidos no centro de pesquisa clínica do Hospital de Clínicas de Porto Alegre (Centro do estudo PREVER e Laboratório de Fisiopatologia do Exercício). A partir de um primeiro contato telefônico, os indivíduos interessados em participar do estudo e que estejam de acordo com os critérios de inclusão e exclusão do estudo serão recebidos pelo pesquisador responsável e pela sua equipe. Confirmada a elegibilidade do participante e o mesmo tirar suas dúvidas sobre os procedimentos que serão adotados, riscos e benefícios do estudo, o participante assinará TCLE.

## Treinamentos aplicados

Os grupos TC2 e TC4 realizarão os treinamento com intensidade e volume progressivos, periodizados de forma linear, e realizarão a mesma sobrecarga semanal de treinamento (i.e., minutos por semana, séries, repetições, carga e exercícios), porém terão essa sobrecarga dividida em diferentes frequências semanais. O treinamento terá a duração de 12 semanas, com volume semanal total de 120 min nas primeiras semanas (semanas 1-6), progredindo para 150 min/semanais nas últimas semanas (semanas 7-12). Sendo assim, os grupos diferenciarão seus treinamentos exclusivamente pelo número de sessões semanais que serão utilizadas para realizar esse treinamento, com um grupo realizando 2 sessões semanais (TC2) e o outro grupo 4 sessões semanais (TC4).

A primeira parte da sessão será composta pelos seguintes ERs: apoio, remada na barra, agachamento, flexão plantar, abdominais e equilíbrio unipodal. A progressão em volume será realizada através do aumento do número de séries e repetições, e/ou pelo tempo (segundos) de uma série. A intensidade do esforço será ajustada a partir da amplitude de movimento dos exercícios, velocidade de execução, e variações no número de apoios para estabilização dos exercícios. Os indivíduos deverão realizar de 1-4 séries de 10-15 repetições com a carga correspondente a 60-70% de 1RM monitorada através de percepção subjetiva de esforço (PSE), monitorada através da escala CR10. Um intervalo de 90-120 segundos será adotado entre as séries e entre os exercícios. A segunda parte da sessão será composta por EA realizado em esteira ergométrica ou em pista de atletismo e terá duração de 20-50 minutos. A intensidade dessas sessões será controlada através da frequência cardíaca de reserva (FC_reserva_) dos indivíduos, utilizando intensidades entre 60-70% da FC_reserva_. Na ausência de monitores de FC será utilizada a escala de percepção de esforço correspondente as intensidades supracitadas, monitorada através da escala de BORG com índices de 6-20. Na última sessão da semana a cada período de 4 semanas, após o ER e o final de cada sessão, serão registradas informações referentes a PSE dos participantes na sessão e ao nível de divertimento ao realizar o treinamento. Convém salientar que as sessões de treinamento sempre iniciarão pelo ER, e posteriormente, realizarão o EA, pois essa parece ser a melhor ordem para realizar o treinamento nessa população[^41^](#_ENREF_41)^,^ [^42^](#_ENREF_42).

## Avaliações

Antes do início do estudo (semana 0) e ao final do período de intervenções (semana 13), os participantes realizarão avaliações referentes a PA, hemoglobina glicada, função endotelial, nível de aptidão física e qualidade de vida. Divididas em 3 visitas e com intervalo entre as visitas de de 24-72h, as diferentes avaliações visam verificar os possíveis efeitos das diferentes intervenções nas variáveis de desfecho do estudo.Um recordatório alimentar será utilizado para caracterizar o comportamento alimentar e avaliar uma possível interferência da alimentação sobre as respostas ao treinamento. Os sujeitos serão recomendados a não alterar seus hábitos alimentares durante o período de estudo.

Na primeira visita, após assinatura do TCLE, será realizada uma anamnese para caracterização da amostra, coleta de medidas antropométricas através de medidas de estatura e a massa corporal, realizadas a partir de um estadiômetro e de uma balança analógica (marca FILIZOLA, BRASIL). Com esses valores serão calculados seus índices de massa corporal (IMC), segundo a equação massa (kg)/estatura(m)². Também será realizado um questionário para avaliação da qualidade de vida dos participantes (WHOQOL-BREF). Ainda nessa sessão, serão realizadas medidas de PA (após 20 minutos de repouso) seguido de uma familiarização com os exercícios que serão utilizados nos testes de força e potência muscular e com a máscara que será utilizada na coleta de gases durante o teste cardiopulmonar.

Na segunda visita, serão realizadas novas medidas de PA e conduzida a avaliação de função endotelial por meio da análise de dilatação mediada pelo fluxo. Também será realizado o eletrocardiograma em repouso e a coleta de sangue para análise dos níveis de hemoglobina glicada. Dessa coleta sanguínea, será utilizada uma amostra para análise do colesterol total, HDL-c, LDL-c, VLDL-c, que servirão para caracterização da amostra. Posteriormente, será feita a colocação do equipamento de monitoramento ambulatorial de pressão arterial (MAPA). Após 24h da segunda visita, cada paciente deverá retornar ao laboratório após 24h para retirada da MAPA.

Na terceira visita, após retirada da MAPA, será conduzida a avaliação dos testes de força e potência muscular, bem como a avaliação cardiorrespiratória e de equilíbrio unipodal. Primeiramente serão conduzidos os testes de força, potência e equilíbrio unipodal e, posteriormente, após um descanso de 10 minutos, será o conduzido o teste cardiopulmonar.

*Mensuração da pressão arterial*

A pressão arterial durante o período de coletas no laboratório será avaliada de acordo com o protocolo de mensuração das diretrizes de hipertensão arterial da Sociedade Brasileira de Hipertensão e Sociedade Brasileira de Nefrologia. Através dessas mensurações os valores de PAS, PAD e PAM serão obtidos pelo método oscilométrico automático (Dinamap, Critikon, EUA). Os resultados do presente estudo terão medidas que estarão sem subjetividade do operador (operador-independente).

A MAPA, caracterizada pela medição periódica de PA em ambiente de atividades diárias, será realizada por aparelho oscilométrico automático portátil (ABP 2400, Mortara, Milwaukee, EUA). Os participantes receberão orientações verbais em relação à realização do exame (funcionamento do aparelho, posição de medição, diário de atividades). Após isso, lhes será vestido após o término de cada sessão experimental, no braço não dominante, o manguito do aparelho de MAPA, configurado para realizar a medida da PA de vigília a cada 15 minutos, e noturna a cada 20 minutos. O período de vigília será considerado das 6:00 às 23h e o período noturno das 23h até as 6h. Todos os participantes terão o início dos monitoramentos agendados para o mesmo horário.

*Hemoglobina glicada*

Para as coletas de dados glicêmicos crônicos, será utilizado o exame laboratorial para avaliar os níveis de hemoglobina glicada (HbA1c), pelo método da Cromatografia Líquida de Alta Performance (HPLC).

*Função Endotelial*

Será realizada por ultrassonografia de alta resolução (HD7XE, Phillips, EUA) da artéria braquial em conjunto com Doppler vascular (para obtenção da velocidade de fluxo), através de um transdutor de altafreqüência (3-12MHz). A avaliação da dilatação mediada pelo fluxo (FMD) será obtida em sala com baixa luminosidade, temperatura controlada e após 15minutos de repouso deitado. Após manobra de oclusão arterial por 5 minutos, o diâmetro será avaliado por 120 segundos e variações serão relativizadas ao diâmetro pré-oclusão arterial.

*Aptidão cardiorrespiratória*

Os participantes do estudo serão submetidos a testes máximos pré e pós-treinamento para mensurar o consumo máximo de oxigênio (VO_2máx_). Os testes serão realizados em esteira ergométrica com máscara e analisadores de gases, que serão previamente ligados e calibrados a fim de evitar qualquer tipo de contratempo. Para realização do teste, será utilizada uma esteira ergométrica modelo 10200 ATL da marca IMBRAMED (Porto Alegre, Brasil), com resolução de velocidade e inclinação de 0,1km.h^-1^ e 1%, respectivamente. Primeiramente será realizado aquecimento durante 3 min sendo a velocidade aumentada lentamente até atingir 3 km/h. Logo após, o teste iniciará a 3-4 km/h e inclinação de 1%, com incrementos na velocidade de 0,5 km/h a cada 30 segundos e na inclinação de 1% a cada dois minutos. O teste será interrompido quando o participante indicar sua exaustão através de um sinal manual. Cabe salientar que haverá a presença de um médico nesta sessão de testes. Os gases respiratórios serão coletados através do analisador de gases portátil do tipo caixa de mistura (VO2000, MedGraphics, Ann Arbor, USA).

*Função neuromuscular*

Para a avaliação da força e potência de membros superiores será realizado o teste de preensão palmar através do uso do equipamento *handgrip* (Marca: Jamar, USA) e arremesso de *medicine ball*, respectivamente. O teste de preensão palmar foi escolhido em função da grande validade externa dessa avaliação e da associação entre níveis de força obtidos nessa avaliação e mortalidade da população^[28](#_ENREF_28)^. Antes do teste será explicado sua finalidade e como segurar o dinamômetro para uma familiarização. Para testar a força máxima de preensão palmar, o avaliado deverá estar sentado numa cadeira sem braços, com as costas apoiadas no encosto, os pés apoiados no chão e a mão que não está sendo avaliada relaxada em cima da coxa do mesmo lado. O avaliador estará sentado à frente do avaliado, sustentando o dinamômetro de maneira que o indivíduo avaliado fique com o ombro aduzido com rotação neutra, o cotovelo permaneça num ângulo de 90 graus e o punho em pegada neutra. Ao sinal do avaliador, o sujeito deverá apertar o dinamômetro com a mão com a maior força possível durante 3 segundos. O procedimento será repetido 3 vezes em cada mão, alternando as mãos dominante e não-dominante, com intervalo mínimo de 1 minuto. O teste de arremesso de *medicine ball,* será realizado 3 tentativas com 20 segundos de intervalo. Para este teste, será utilizada uma medicine ball de 1 kg para mulheres e de 2 kg para homens. A partir de uma posição de ombros fletidos a 90 graus, o avaliado lançará a bola o mais longe possível, realizando o movimento em velocidade máxima a fim de uma maior eficiência do ciclo alongamento-encurtamento.

Para avaliar a força e potência muscular dos membros inferiores será utilizado o teste de sentar e levantar e o salto vertical *Countermovement Jump* (CMJ), respectivamente. A avaliação da potência muscular de membros inferiores será obtida através da altura do salto CMJ vertical monitorado através de um *software* (My Jump 2)^43^. Antes de iniciar a coleta de dados os sujeitos realizarão um aquecimento que servirá também como uma familiarização com o teste, realizando 3-5 saltos. Os sujeitos partirão da posição ortostática, com as mãos postadas na cintura, realizarão um agachamento e posteriormente a fase de voo. Para a correta execução do teste, os participantes serão instruídos a manterem as mãos postadas na cintura durante todo o teste, realizar a fase de transição entre o agachamento e a fase de voo rapidamente, saltar o mais alto possível e aterrissar no mesmo ponto de partida. Durante o teste, serão realizados 3 saltos CMJ com um intervalo de 20 segundos entre cada salto. A avalição de força dos membros inferiores será obtida através da realização do número máximo de agachamentos que os participantes conseguem realizar durante 30 segundos. Além disso, será contabilizado o tempo (segundos) que os participantes levam para realizar os primeiros cinco agachamentos. O teste será inciado com o participante sentado na cadeira, as costas não deverão estar apoiadas no encontos. Pés afastados à largura dos ombros e totalmente apoiados no solo. Os membros superiores estão cruzados ao nível dos pulsos e contra o peito. Ao sinal de “partida” o participante eleva-se até à extensão máxima (posição vertical) e regressa à posição inicial sentado.

*Avaliação da qualidade de vida*

Para avaliar a qualidade de vida será utilizado o questionário WHOQOL-BREF, versão traduzida e validada no Brasil. O questionário contém 26 questões e é dividido em quatro domínios (físico, psicológico, social e meio ambiente). As respostas seguem uma escala de Linkert (1 a 5, quanto maior a pontuação melhor a qualidade de vida) e os valores de cada domínio serão expressos em valores percentuais.

## Randomização e sigilo de alocação

Para a randomização dos participantes nos diferentes grupos experimentais (TC2 e TC4) será utilizada a randomização estratificada e em blocos. Os estratos serão constituídos por três faixas de idade (50-59, 60-69 e 70-79 anos). Dessa forma, os participantes nas diferentes faixas etárias estarão distribuídos de forma balanceada por estrato de idade e aleatoriamente entre as diferentes intervenções. Ainda, serão criados blocos de distribuição dos participantes entre as intervenções para garantir que o número de participantes em cada grupo seja igual.

Os sujeitos e os pesquisadores responsáveis pelas intervenções estarão cegados para alocação das intervenções e só terão acesso a essa informação no momento da randomização. O estudo contará com um epidemiologista responsável somente pela randomização e sigilo de alocação, não participando do recrutamento, avaliação ou intervenção com os pacientes. Este pesquisador irá informar através de contato telefônico com os pesquisadores executores as diferentes sequências de randomização para cada sujeito da pesquisa. O pesquisador responsável pela análise estatística das variáveis de desfecho do estudo também será cegado através de diferentes códigos para cada tipo de intervenção. O processo de randomização será realizado em software on-line ([randomization.com](http://www.randomization.com)).

No caso de uma disponibilidade de participantes maior do que o número necessário será convidado os primeiros participantes que entrarem em contato e preencherem todos os pré-requisitos para participação no estudo. Por outro lado, caso a disponibilidade seja menor do que a de participantes elegíveis, a divulgação do projeto seguirá até que este número amostral necessário seja atingido.

## Considerações éticas

O presente projeto de estudo será concebido de acordo com as condutas éticas estabelecidas na Resolução 466/2012, do Conselho Nacional de Saúde. Os participantes terão sua cópia do TCLE, o qual será esclarecido por um membro da equipe de pesquisadores, antes do início de qualquer procedimento no estudo. O projeto será conduzido após aprovação pelo Comitê de Ética ao qual o projeto será designado através da Plataforma Brasil.

Durante o período de intervenção, se for relatado alguma dor ou desconforto osteomuscular ou algum mal-estar durante a realização do treinamento o mesmo será interrompido imediatamente. Caso haja necessidade de atendimento ou procedimento que estejam além das competências do pesquisador, os mesmos poderão ser realizados pela Equipe de Enfermagem do Centro de Pesquisas Clínicas (ramal 6324) ou mesmo pelo Serviço de Emergência da instituição. Neste caso, o pesquisador entrará em contato com o esse pelo ramal 8653 e solicitar contato com o médico regulador, explicar a situação e acompanhar o paciente até à Emergência. O deslocamento do indivíduo será feito por cadeira de rodas, maca ou ambulância. Além disso, os pesquisadores responsáveis entrarão em contato com algum familiar ou pessoa próxima (relatado pelo participante na primeira visita), para informar sobre o ocorrido.

## Análise estatística

Para analisar os dados coletados será utilizada primeiramente a estatística descritiva. Serão utilizados os testes de Shapiro-Wilk e Levene para a normalidade dos dados e homogeneidade das variâncias, respectivamente. Se os dados apresentarem distribuição normal e homogênea, utilizar-se-á a estatística paramétrica. Serão realizadas transformações caso os dados não apresentem distribuição normal para torná-los paramétricos. A *Generalized Estimating Equations* (GEE) e o teste *post-hoc* de Bonferroni serão utilizados para a comparação entre os momentos e grupos/ intervenções. Ainda, será realizada a análise estatística por protocolo, na qual serão excluídos das análises aqueles que tiverem apresentado três faltas consecutivas nos treinamentos, assim como os que obtiverem uma frequência inferior que 75% durante o período das intervenções. Também será realizada análise estatística por intenção de tratar, na qual todos os participantes randomizados serão incluídos nas análises. O nível de significância adotado será α = 0,05 para todas as análises. Para a execução dos procedimentos estatísticos, será utilizado o pacote estatístico SPSS versão 22.0.

# CRONOGRAMA

|  | 2019/2 | 2020/1 | 2020/2 | 2021/1 |
| --- | --- | --- | --- | --- |
| Submissão do projeto ao comitê de ética | x |  |  |  |
| Treinamento da equipe de trabalho | x |  |  |  |
| Recrutamento de participantes | x | x | x |  |
| Avaliações e intervenções | x | x | x | x |
| Análise e discussão de resultados |  |  |  | x |
| Redação e submissão de artigo científico |  |  |  | x |

# ORÇAMENTO

Materiais permanentes como aparelhos de pressão, aparelhos para as sessões de exercício, bem como outros materiais permanentes necessários para as avaliações do projeto já estão disponíveis no Laboratório de Fisiopatologia do Exercício do Hospital de Clínicas de Porto Alegre e no centro do estudo PREVER.

|  | Quantidade | Valor Unitário | Valor Total |
| --- | --- | --- | --- |
| Folhas A4 (pacote) | 5 | R$ 13,00 | R$ 65,00 |
| Gel para ultrassom (300g) | 10 | R$3,24 | R$ 32,40 |
| Eletrodo descartável | 1000 | R$ 0,63 | R$ 360,00 |
| Eletrocardiograma em repouso | 98 | R$ 5,15 | R$ 504,70 |
| Hemoglobina glicada (sangue) | 196 | R$ 10,17 | R$ 1993,32 |
| Colesterol total (sangue) | 196 | R$ 1,85 | R$ 362,60 |
| Ergoespirometria | 98 | R$ 30,00 | R$ 2940,00 |
| **TOTAL** |  |  | **R$ 6.258,02** |

# REFERÊNCIAS

**1.** Ferrari R, Kruel LF, Cadore EL, et al. Efficiency of twice weekly concurrent training in trained elderly men. *Experimental gerontology*. 2013; 48: 1236-42.

**2.** Ferrari R, Fuchs SC, Kruel LF, et al. Effects of Different Concurrent Resistance and Aerobic Training Frequencies on Muscle Power and Muscle Quality in Trained Elderly Men: A Randomized Clinical Trial. *Aging and disease*. 2016; 7: 697-704.

**3.** Ferrari R, Umpierre D, Vogel G, et al. Effects of concurrent and aerobic exercises on postexercise hypotension in elderly hypertensive men. *Experimental gerontology*. 2017; 98: 1-7.

**4.** da Costa JS, Barcellos FC, Sclowitz ML, et al. Hypertension prevalence and its associated risk factors in adults: a population-based study in Pelotas. *Arq Bras Cardiol*. 2007; 88: 59-65.

**5.** Moreira LB, Fuchs SC, Wiehe M, Gus M, Moraes RS and Fuchs FD. Incidence of hypertension in Porto Alegre, Brazil: a population-based study. *J Hum Hypertens*. 2008; 22: 48-50.

**6.** Whelton PK, Carey RM, Aronow WS, et al. 2017 ACC/AHA/AAPA/ABC/ACPM/AGS/APhA/ASH/ASPC/NMA/PCNA Guideline for the Prevention, Detection, Evaluation, and Management of High Blood Pressure in Adults: A Report of the American College of Cardiology/American Heart Association Task Force on Clinical Practice Guidelines. *J Am Coll Cardiol*. 2017.

**7.** Bundy JD, Li C, Stuchlik P, et al. Systolic Blood Pressure Reduction and Risk of Cardiovascular Disease and Mortality: A Systematic Review and Network Meta-analysis. *JAMA Cardiol*. 2017; 2: 775-81.

**8.** Burnier M, Oparil S, Narkiewicz K and Kjeldsen SE. New 2017 American Heart Association and American College of Cardiology guideline for hypertension in the adults: major paradigm shifts, but will they help to fight against the hypertension disease burden? *Blood Press*. 2018; 27: 62-5.

**9.** Oparil S, Acelajado MC, Bakris GL, et al. Hypertension. *Nat Rev Dis Primers*. 2018; 4: 18014.

**10.** Lewington S, Clarke R, Qizilbash N, Peto R, Collins R and Prospective Studies C. Age-specific relevance of usual blood pressure to vascular mortality: a meta-analysis of individual data for one million adults in 61 prospective studies. *Lancet*. 2002; 360: 1903-13.

**11.** Picon RV, Fuchs FD, Moreira LB, Riegel G and Fuchs SC. Trends in prevalence of hypertension in Brazil: a systematic review with meta-analysis. *PloS one*. 2012; 7: e48255.

**12.** Picon RV, Fuchs FD, Moreira LB and Fuchs SC. Prevalence of hypertension among elderly persons in urban Brazil: a systematic review with meta-analysis. *American journal of hypertension*. 2013; 26: 541-8.

**13.** Ishiguro H, Kodama S, Horikawa C, et al. In Search of the Ideal Resistance Training Program to Improve Glycemic Control and its Indication for Patients with Type 2 Diabetes Mellitus: A Systematic Review and Meta-Analysis. *Sports medicine*. 2016; 46: 67-77.

**14.** Delevatti RS, Kanitz AC, Alberton CL, et al. Glucose control can be similarly improved after aquatic or dry-land aerobic training in patients with type 2 diabetes: A randomized clinical trial. *J Sci Med Sport*. 2016; 19: 688-93.

**15.** Kirkman MS, Briscoe VJ, Clark N, et al. Diabetes in older adults. *Diabetes Care*. 2012; 35: 2650-64.

**16.** Fuchs FD, Gus M, Moreira WD, et al. Blood pressure effects of antihypertensive drugs and changes in lifestyle in a Brazilian hypertensive cohort. *Journal of hypertension*. 1997; 15: 783-92.

**17.** Gus I, Harzheim E, Zaslavsky C, Medina C and Gus M. Prevalence, awareness, and control of systemic arterial hypertension in the state of Rio Grande do Sul. *Arquivos brasileiros de cardiologia*. 2004; 83: 429-33; 4-8.

**18.** Sociedade Brasileira de C, Sociedade Brasileira de H and Sociedade Brasileira de N. [VI Brazilian Guidelines on Hypertension]. *Arquivos brasileiros de cardiologia*. 2010; 95: 1-51.

**19.** Sillanpaa E, Hakkinen A, Nyman K, et al. Body composition and fitness during strength and/or endurance training in older men. *Medicine and science in sports and exercise*. 2008; 40: 950-8.

**20.** Sillanpaa E, Hakkinen A, Punnonen K, Hakkinen K and Laaksonen DE. Effects of strength and endurance training on metabolic risk factors in healthy 40-65-year-old men. *Scandinavian journal of medicine & science in sports*. 2009; 19: 885-95.

**21.** Moraes MR, Bacurau RF, Casarini DE, et al. Chronic conventional resistance exercise reduces blood pressure in stage 1 hypertensive men. *Journal of strength and conditioning research / National Strength & Conditioning Association*. 2012; 26: 1122-9.

**22.** Cornelissen VA, Buys R and Smart NA. Endurance exercise beneficially affects ambulatory blood pressure: a systematic review and meta-analysis. *Journal of hypertension*. 2013; 31: 639-48.

**23.** Millar PJ, McGowan CL, Cornelissen VA, Araujo CG and Swaine IL. Evidence for the role of isometric exercise training in reducing blood pressure: potential mechanisms and future directions. *Sports medicine*. 2014; 44: 345-56.

**24.** Cornelissen VA and Smart NA. Exercise training for blood pressure: a systematic review and meta-analysis. *Journal of the American Heart Association*. 2013; 2: e004473.

**25.** Kokkinos P, Doumas M, Myers J, et al. A graded association of exercise capacity and all-cause mortality in males with high-normal blood pressure. *Blood pressure*. 2009; 18: 261-7.

**26.** Blair SN, Kampert JB, Kohl HW, 3rd, et al. Influences of cardiorespiratory fitness and other precursors on cardiovascular disease and all-cause mortality in men and women. *Jama*. 1996; 276: 205-10.

**27.** Lee DC, Artero EG, Sui X and Blair SN. Mortality trends in the general population: the importance of cardiorespiratory fitness. *Journal of psychopharmacology*. 2010; 24: 27-35.

**28.** Ruiz JR, Sui X, Lobelo F, et al. Association between muscular strength and mortality in men: prospective cohort study. *Bmj*. 2008; 337: a439.

**29.** MacDonald HV, Johnson BT, Huedo-Medina TB, et al. Dynamic Resistance Training as Stand-Alone Antihypertensive Lifestyle Therapy: A Meta-Analysis. *Journal of the American Heart Association*. 2016; 5.

**30.** American College of Sports M, Chodzko-Zajko WJ, Proctor DN, et al. American College of Sports Medicine position stand. Exercise and physical activity for older adults. *Medicine and science in sports and exercise*. 2009; 41: 1510-30.

**31.** Sayer AA, Robinson SM, Patel HP, Shavlakadze T, Cooper C and Grounds MD. New horizons in the pathogenesis, diagnosis and management of sarcopenia. *Age and ageing*. 2013; 42: 145-50.

**32.** Reid KF and Fielding RA. Skeletal muscle power: a critical determinant of physical functioning in older adults. *Exercise and sport sciences reviews*. 2012; 40: 4-12.

**33.** Hagberg JM, Montain SJ and Martin WH, 3rd. Blood pressure and hemodynamic responses after exercise in older hypertensives. *Journal of applied physiology*. 1987; 63: 270-6.

**34.** Rezk CC, Marrache RC, Tinucci T, Mion D, Jr. and Forjaz CL. Post-resistance exercise hypotension, hemodynamics, and heart rate variability: influence of exercise intensity. *European journal of applied physiology*. 2006; 98: 105-12.

**35.** Brito LC, Queiroz AC and Forjaz CL. Influence of population and exercise protocol characteristics on hemodynamic determinants of post-aerobic exercise hypotension. *Brazilian journal of medical and biological research.*2014; 47: 626-36.

**36.** MacDonald JR. Potential causes, mechanisms, and implications of post exercise hypotension. *Journal of human hypertension*. 2002; 16: 225-36.

**37.** Queiroz AC, Sousa JC, Cavalli AA, et al. Post-resistance exercise hemodynamic and autonomic responses: Comparison between normotensive and hypertensive men. *Scandinavian journal of medicine & science in sports*. 2015; 25: 486-94.

**38.** Queiroz AC, Rezk CC, Teixeira L, Tinucci T, Mion D and Forjaz CL. Gender influence on post-resistance exercise hypotension and hemodynamics. *International journal of sports medicine*. 2013; 34: 939-44.

**39.** Borjesson M, Onerup A, Lundqvist S and Dahlof B. Physical activity and exercise lower blood pressure in individuals with hypertension: narrative review of 27 RCTs. *Br J Sports Med*. 2016; 50: 356-61.

**40.** Piercy KL, Troiano RP, Ballard RM, et al. The Physical Activity Guidelines for Americans. *Jama*. 2018; 320: 2020-8.

**41.** Cadore EL, Izquierdo M, Alberton CL, et al. Strength prior to endurance intra-session exercise sequence optimizes neuromuscular and cardiovascular gains in elderly men. *Experimental gerontology*. 2012; 47: 164-9.

**42.** Pinto SS, Alberton CL, Bagatini NC, et al. Neuromuscular adaptations to water-based concurrent training in postmenopausal women: effects of intrasession exercise sequence. *Age*. 2015; 37: 9751.

**43.** Balsalobre-Fernández, Carlos et al. “The validity and reliability of an iPhone app for measuring vertical jump performance.” Journal of sports sciences 33 15 (2015): 1574-9.

**ANEXO I – TERMO DE CONSENTIMENTO LIVRE E ESCLARECIDO**

**Título do projeto:** Comparação de diferentes frequências semanais de treinamento combinado na pressão arterial e outros fatores de risco cardiovasculares em indivíduos com hipertensão: um ensaio clínico randomizado

A prática regular de treinamento físico é uma importante forma de beneficiar a saúde de pessoas saudáveis ou doentes. As entidades de saúde recomendam a prática de atividade física para prevenção e como parte do tratamento da hipertensão, podendo ainda proporcional uma melhora na qualidade de vida dos praticantes.

Você está sendo convidado (a) a participar de uma pesquisa cujo objetivo é avaliar os efeitos do treinamento combinado (exercícios de fortalecimento muscular e exercício de caminhada ou corrida) na redução da pressão arterial de 24 horas (MAPA). Todos os participantes realizarão 12 semanas de treinamento combinado com o mesmo volume semanal de treinamento (ou seja, mesma quantidade de minutos por semana). Entretanto, serão divididos de forma aleatória quanto ao número de sessões semanais que terão para completar esse volume. Metade dos participantes realizará exercício duas vezes na semana e a outra metade quatro vezes na semana.

Essa pesquisa está sendo realizada pelo Laboratório de Fisiopatologia do Exercício do Centro de Pesquisa Clínica do Hospital de Clínicas de Porto Alegre (HCPA) em parceria com o centro do estudo PREVER, ambos localizados no Hospital de Clínicas de Porto Alegre, RS.

Se você aceitar participar da pesquisa, os procedimentos envolvidos em sua participação são os seguintes:

Avaliações

Visita 1

- Será medida a sua altura, massa corporal e circunferência da cintura.

- Será realizada a medição da sua pressão arterial através de um monitor automático.

- Será realizada uma coleta sanguínea para avaliar os níveis de hemoglobina glicada e colesterol total.

- Será realizado um eletrocardiograma de repouso.

Esta visita será realizada no Centro de Pesquisa Clínica do HCPA terá duração de 2 horas.

Visita 2

- Será feita a avaliação da dilatação da artéria braquial através de exame de imagem (ultrassonografia). Além disso, colocaremos o equipamento de monitoramento ambulatorial de pressão arterial (MAPA), durante 24 horas. Você deverá retornar ao laboratório após 24 horas para retirada do equipamento.

Esta visita terá duração de 30 minutos.

Visita 3

- Retirada da MAPA.

- Serão realizadas avaliações cardiorrespiratória, força e potência muscular e equilíbrio em um pé. Você poderá sentir cansaço nos minutos seguintes aos testes, dores moderadas e/ou fadiga musculares nas pernas durante as 24 -72h posteriores aos testes.

Semana 1-12

Após as avaliações preliminares, você será sorteado para integrar um dos grupos de treinamento do estudo:

Grupo 1: Treinamento combinado duas vezes na semana - Neste grupo os participantes realização treinos de fortalecimento muscular, utilizando o peso corporal como sobrecarga; treinamento aeróbico, caminhada ou corrida realizado em esteira com duração de 40 minutos por sessão evoluindo até 50 minutos por sessão ao final do estudo.

Grupo 2: Treinamento combinado quatro vezes na semana - Os participantes desse grupo realização treinamento de fortalecimento muscular e aeróbico, conforme descrito acima, com duração de 20 minutos por sessão evoluindo até 25 minutos por sessão ao final do estudo.

O programa de treinamento combinado consiste de exercícios de força e caminhada/ corrida, com diferentes frequências semanais (duas vezes ou quatro vezes na semana), durante 12 semanas. Os treinamentos serão no laboratório de exercício físico do Hospital de Clínicas de Porto Alegre, onde você será acompanhado (a) por profissionais de educação física e/ou médicos. As sessões iniciarão com os exercícios de força: apoio, remada na barra, agachamento, abdominais e equilíbrio em um pé; após será realizado o exercício de caminhada e/ou corrida. Após a execução dos programas de acompanhamento, serão repetidas as mesmas avaliações realizadas nas visitas iniciais. Ou seja, você deverá retornar ao Centro de Pesquisa Clínica do HCPA para mais 3 visitas, nas quais os procedimentos descritos acima (Visitas 1-3) serão repetidos.

Você poderá ou não se beneficiar com a participação na pesquisa. Espera-se que ambos os programas possam alterar de maneira positiva o seu perfil de pressão arterial, porém isso pode não ser verdade para todos os que participarem do estudo, pois há grande variação entre as respostas de cada um. Contudo, o estudo das respostas dos participantes aos programas pode trazer uma contribuição ao entendimento da hipertensão na população.

Teremos medidas de segurança e procedimentos para prevenção de riscos durante os procedimentos da pesquisa. Em caso de emergência, o serviço médico será imediatamente contatado. Os pesquisadores darão assistência de primeiros socorros, e a pessoa que você informou para o caso de emergência será avisada. Caso ocorra alguma intercorrência ou dano, resultante de sua participação na pesquisa, você receberá todo o atendimento necessário, sem nenhum custo pessoal. Caso surjam quaisquer informações novas que inviabilizem, prejudiquem ou modifiquem sua participação no estudo, você será avisado com antecedência. No caso de aparecimento de anormalidades em quaisquer dos exames realizados, você será avisado e aconselhado a buscar acompanhamento médico.

Sua participação na pesquisa é totalmente voluntária, ou seja, não é obrigatória. Caso você decida não participar, ou ainda, desistir de participar e retirar seu consentimento, não haverá nenhum prejuízo ao atendimento que você recebe ou possa vir a receber na instituição. Mudanças de qualquer natureza nos procedimentos do estudo lhe serão informadas antes de ocorrerem e lhe será dada toda a autonomia para decidir sua permanência no mesmo.

Não está previsto nenhum tipo de pagamento pela sua participação na pesquisa e você não terá nenhum custo com respeito aos procedimentos envolvidos. As informações obtidas a partir de sua participação serão tratadas anonimamente. Os dados estarão disponíveis para o participante e para quem você autorizar, e poderão ser utilizados anonimamente para fins acadêmicos científicos.

Se você tiver dúvidas, faça as perguntas que desejar antes de decidir sua participação. Caso você tenha dúvidas, poderá entrar em contato com o pesquisador responsável, Prof. Dr. Rodrigo Ferrari da Silva pelo telefone (51) 999012660, ou com o Comitê de Ética em Pesquisa do Hospital de Clínicas de Porto Alegre (HCPA), pelo telefone (51) 33597640, ou no 2° andar do HCPA, sala 2227, de segunda à sexta, das 8h às 17h.

Esse Termo é assinado em duas vias, sendo uma para o participante e outra para os pesquisadores.

____________________________________(Nome do participante da pesquisa)

____________________________________(Assinatura)

____________________________________(Nome do pesquisador que aplicou o Termo)

____________________________________(Assinatura)

Porto Alegre, ______de____________________de____________.
